# Differential associations of beverage consumption with diabetic retinopathy: a systematic review and dose-response meta-analysis of observational studies

**DOI:** 10.3389/fmed.2026.1819832

**Published:** 2026-06-17

**Authors:** Siyan Liu, Xuan Wang, Guojun Chao, Wei Lu, Qi Wu, Yuxin Lv, Shuangshuang Yi, Zhengzheng Wu, Jing Yan

**Affiliations:** 1 China Academy of Chinese Medical Sciences Eye Hospital, Beijing, China; 2 Anshan Hospital of Traditional Chinese Medicine, Anshan, Liaoning, China; 3 Yanqing Hospital of Beijing Chinese Medicine Hospital, Beijing, China

**Keywords:** diabetic retinopathy, beverages, alcohol, coffee, tea, artificially sweetened beverages, dose-response relationship, meta-analysis

## Abstract

**Background:**

Diabetic retinopathy (DR) is a major blinding microvascular complication of diabetes mellitus. The role of dietary factors in its development has garnered increasing attention. However, the association between the consumption of various common beverages and DR risk remains unclear.

**Objective:**

Systematically evaluate the association and dose-response relationship between the intake of alcohol, wine, spirits, sugary drinks (SSBs), artificially sweetened drinks (ASBs), coffee, tea, natural fruit juice and yogurt with the risk of DR.

**Methods:**

The systematic literature search was conducted across four major electronic databases—PubMed, Embase, Web of Science, and the Cochrane Library—from their inception dates through February 15, 2026. Fixed- or random-effects models were used to pool effect sizes. Linear and non-linear dose-response relationships were analyzed using a two-stage dose-response meta-analysis. Study quality was assessed using the Newcastle-Ottawa Scale.

**Results:**

A total of 25 studies (94,119 participants) were included, with 84% being of high quality. Overall, no significant association was found between alcohol consumption and DR. However, heterogeneity across disease stages was observed: alcohol was inversely associated with non-proliferative diabetic retinopathy (NPDR) but positively associated with vision-threatening diabetic retinopathy (VTDR). In type 1 diabetes, alcohol showed a non-linear inverse association (OR = 0.54, 95%CI 0.37–0.80 at 50 g/week). Tea consumption was significantly inversely associated with DR (HR = 0.72, 95%CI 0.58–0.90 at 295 g/week). Coffee exhibited an inverse association only at a high dose of 4,032 g/week (OR = 0.42, 95%CI 0.26–0.68). High consumption of ASBs (1,420 g/week) significantly increased the risk of proliferative diabetic retinopathy (PDR) (OR = 2.62, 95%CI 1.14–6.04). No significant associations were found for SSBs, natural fruit juice, or yogurt.

**Conclusion:**

The association between alcohol and DR varies by disease stage and beverage type. Tea and high-dose coffee consumption suggest non-linear inverse associations against DR, while high-dose ASB intake may specifically increase the risk of PDR. Given that several analyses were based on a limited number of observational studies, further prospective studies are needed to refine these assessments and provide evidence for dietary prevention strategies.

**Systematic review registration:**

Registration number: CRD420261308253. https://www.crd.york.ac.uk/PROSPERO/view/CRD420261308253.

## 1 Introduction

Diabetic retinopathy (DR) is the leading cause of vision loss among working-age adults worldwide, affecting approximately one-third of individuals with diabetes mellitus (1). Although optimizing blood sugar, blood pressure and lipid management is the cornerstone for delaying the progression of diabetic retinopathy, lifestyle factors such as diet have become important supplementary intervention targets.

As integral components of daily diet, the health effects of beverages have garnered considerable attention. Evidence regarding alcohol consumption and DR has been inconsistent. While early observational studies failed to identify a clear association, recent research suggests that the relationship may depend on the type of alcohol and drinking pattern (1–3). Coffee and tea are rich in polyphenolic antioxidants, and epidemiological studies suggest that they may be associated with a lower risk of type 2 diabetes (4, 5).

However, studies directly investigating their association with DR are scarce and have yielded inconsistent conclusions. Sugar-sweetened beverages, high in added sugars, are well-established risk factors for obesity, type 2 diabetes, and metabolic syndrome (6–8)—all of which are established risk factors for DR. Artificially sweetened beverages, commonly consumed as substitutes for SSBs, have a controversial impact on metabolic health and vascular complications (7, 9). Natural fruit juice may increase the initial risk of type 2 diabetes and cardiovascular complications by affecting metabolism and cardiovascular health (10, 11). Yogurt, as a fermented dairy product, may exert beneficial metabolic effects through probiotics and calcium content (10). However, direct epidemiological evidence linking these beverages to DR is extremely limited. It should be noted that the above evidence is largely derived from observational studies, and the reported associations are not equivalent to causal effects.

Although previous meta-analyses have examined the association between alcohol and DR, these studies have focused only on single specific beverages without integrating and comparing multiple common drink types (3, 12). The present study included multiple beverages within a single analytical framework based on the following consideration: different beverages are often co-consumed in population diets, and analyzing a single beverage while ignoring the intake of others may subject effect estimates to confounding. Within a mutually adjusted analytical framework, the independent association of each beverage with DR can be assessed, enabling effect estimates for different beverages to be compared. Furthermore, in terms of analytical methods, traditional meta-analyses have typically employed a dichotomous strategy comparing the highest versus lowest intake levels. While this approach can roughly determine whether an exposure is associated with an outcome, it cannot capture the dynamic trend of risk with continuous changes in intake dose. The dose-response meta-analysis applied in the present study addresses this gap. Its core advantage lies in fully utilizing data from multiple exposure categories within original studies to plot continuous exposure-response curves, thereby enabling a more precise quantification of how DR risk changes with beverage intake. This approach not only tests for linear trends but also fits non-linear models, revealing inverse or positive associations that may only manifest within specific dose ranges.

Therefore, this study aims to systematically review and synthesize existing evidence from observational studies on the associations between risk of developing DR and the consumption of alcohol, wine, spirits, SSBs, ASBs, coffee, tea, natural fruit juice, yogurt. Through dose-response meta-analysis, we seek to quantify the shape of the relationship between intake levels of various beverages and DR risk, to evaluate the overall quality and certainty of the current evidence, thereby providing a basis for clinical dietary guidance and directions for future research.

## 2 Methods

The protocol for this systematic review was registered with the International Prospective Register of Systematic Reviews [Registration Number: CRD420261308253, https://www.crd.york.ac.uk/PROSPERO/view/CRD420261308253].

### 2.1 Literature search strategy

Two reviewers (Siyan Liu and Xuan Wang) independently conducted a systematic search of four electronic databases: PubMed, Embase, Web of Science, and the Cochrane Library. The search period covered from the inception of each database to February 15, 2026. The search strategy combined Medical Subject Headings terms and free-text keywords, with core search terms encompassing various beverage names and terms related to diabetic retinopathy. Additionally, the reference lists of included studies were manually searched to identify potentially missed articles. The complete search strategy is provided in the Supplementary material.

### 2.2 Inclusion criteria

The PICOS framework was used to define the inclusion criteria, as detailed below.

**Table 1:** 

Criteria	Explanation
Population	Cohort studies: Participants with diabetes (type 1, type 2, or mixed) but without DR at baseline. Case–control studies: Cases were patients with type 1 or type 2 DR; controls were diabetic patients without DR during the same period. Cross-sectional studies: Included patients with type 1 or type 2 DR.
Intervention/Exposure	Consumption of one or more target beverages, assessed by tools such as food frequency questionnaires, dietary records, 24-h recalls, or interviews.
Comparator	Participants with the lowest intake level of the same beverage (e.g., non-drinkers or those in the lowest intake category).
Outcomes	For cohort studies, the outcome was incident cases of diabetic retinopathy. For cross-sectional and case–control studies, the outcome was prevalent cases of diabetic retinopathy. All cases had to be diagnosed through specialized ophthalmologic examinations, covering any stage of the disease.
Study Design	Observational studies, including cohort studies, case–control studies, and cross-sectional studies, providing risk estimates (OR/RR/HR) with 95% confidence intervals (CIs), or providing raw data allowing for the calculation of these values.

### 2.3 Exclusion criteria

Exclusion criteria encompass review articles, meta-analyses, conference abstracts, editorials, case reports, preclinical studies (including animal experiments and in vitro cell-based investigations), and studies lacking methodologically sound or analyzable outcome data.

Studies that did not involve diabetic patients or included patients with other specific ocular diseases that could affect DR assessment.

Studies that did not provide risk estimates with 95%CIs, and for which these could not be calculated from the raw data.

Duplicate publications; for duplicate reports, only the most comprehensive or most recent study was included.

### 2.4 Study selection and data extraction

EndNote reference management software was used to remove duplicate records. Two reviewers (Siyan Liu and Xuan Wang) independently performed title, abstract screening and full-text review. Disagreements were resolved through discussion or by consulting a third senior researcher (Zhengzheng Wu).

A standardized pre-designed form was used for data extraction. Extracted information included: first author, publication year, country, study design, sample size, participant characteristics, beverage type and assessment method, exposure comparison categories, diagnostic method and definition of DR, adjusted covariates, and the fully adjusted effect estimates with their 95% confidence intervals.

### 2.5 Statistical analysis

Stata version 12.0 was used to calculate pooled odds ratios (ORs) and 95% confidence intervals (CIs) for factors associated with diabetic retinopathy. Heterogeneity was assessed using the *I*^2^ statistic. If *I*^2^ ≤ 50% and *p* > 0.05, indicating low or no heterogeneity, a fixed-effects model was used. Conversely, if *I*^2^ > 50% and *p* ≤ 0.05, indicating significant heterogeneity, a random-effects model was used. In such cases, further subgroup analyses and meta-regression were conducted to explore potential sources of heterogeneity. Publication bias was assessed using funnel plots and Egger’s test for analyses including 10 or more studies, and sensitivity analysis was performed using the leave-one-out method.

Dose-response meta-analysis was performed using Stata version 15.0. The midpoint of each exposure category was assigned as the corresponding dose (e.g., for an alcohol intake category of 2–21 mol/week, the midpoint was 11.5 mol/week). For open-ended lowest categories, the midpoint was set as half the upper bound (e.g., for ≤1 mol/week, the midpoint was 0.5 mol/week). For open-ended highest categories, the dose was estimated as 1.2 times the lower bound (e.g., for ≥22 mol/week, the dose was 26.4 mol/week). Generalized least squares trend estimation (GLST) was used, and restricted cubic splines with three knots (at 10, 50, and 90% percentiles) or four knots (at 5, 35, 65, and 95% percentiles) were fitted to model potential non-linear relationships.

The included studies encompassed cohort studies, case–control studies, and cross-sectional studies. The following principles were applied when pooling: any occurrence of DR, regardless of stage, was combined in the main analysis; the analysis of DR progression included only cohort studies; different DR stages were pooled separately and never merged across stages; and subgroup analyses stratified by study design were performed to assess the impact of design type on pooled effect estimates.

When odds ratios (ORs) and relative risks (RRs) were both included in the same analysis, conversion was performed using the formula RR = OR/(1-p_0_ + p_0_ × OR), where p₀ is the baseline DR risk in the reference group of each study (13). When the baseline risk in the reference group was below 10%, the OR was treated as an approximation of the RR without conversion. Hazard ratios (HRs) were not subjected to this conversion and were analyzed separately.

## 3 Results

### 3.1 Literature screening process

**Figure 1:**
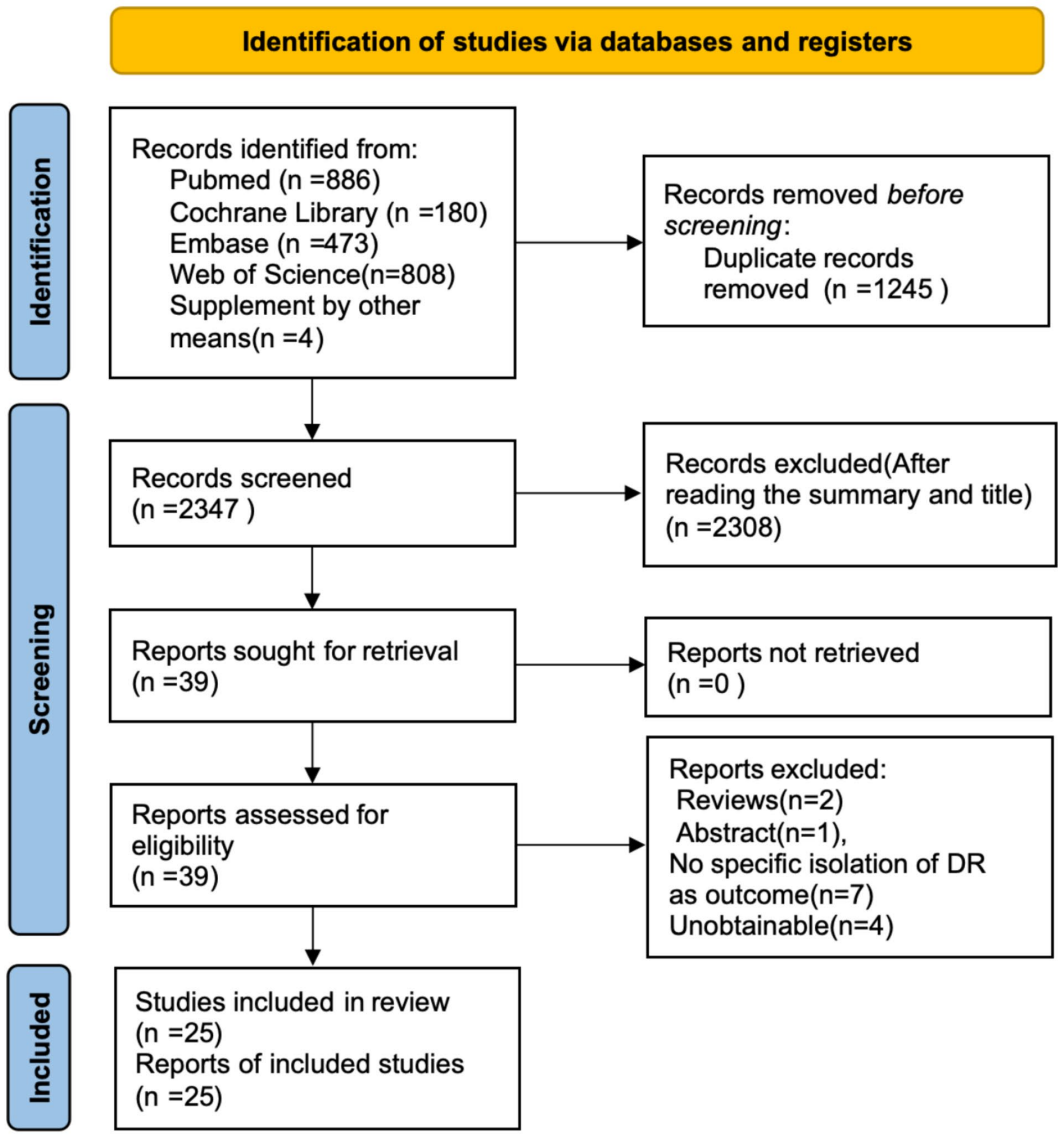
Flow chart of literature screening.

The systematic search yielded 3,592 records. After manual supplement and removal of duplicates, 2,347 titles and abstracts were screened. Thirty-nine full-text articles were reviewed, and ultimately, 25 independent studies were included in this systematic review and meta-analysis (1, 2, 14–36). The literature screening process is detailed in Figure 1. Study designs included prospective cohort studies, cross-sectional studies, and case–control studies. This main text focuses on core analytical results, presenting only the forest plots and dose-response relationship figures for alcohol and various beverages with DR. Detailed characteristics of the included studies, risk of bias assessments, and relevant data are provided in the Supplementary material to ensure the main text is concise while maintaining complete traceability of information.

### 3.2 Characteristics of included studies

The basic characteristics of the 25 studies are summarized in Supplementary Table 1. These studies were published between 1984 and 2025, involving 94,119 unique participants. This included 6 prospective cohort studies, 4 case–control studies, and 15 cross-sectional studies. The study populations covered individuals with type 1 diabetes, type 2 diabetes, and mixed diabetes types, conducted across Asia, Europe, Australia, and the Americas. The follow-up duration for cohort studies ranged from 4.3 to 11.7 years.

Information on beverage intake was primarily collected using validated food frequency questionnaires or self-administered questionnaires; some studies used face-to-face interviews or electronic health records. Exposures included alcohol, coffee, tea, sugar-sweetened beverages, artificially sweetened beverages, fruit juice, and yogurt, with exposure definitions varying across studies regarding categorization.

Regarding confounder adjustment, all studies adjusted for potential confounders in multivariable models. Age was consistently adjusted for. The majority of studies also adjusted for body mass index, glycated hemoglobin, fasting blood glucose, diabetes duration, smoking status, and blood pressure—variables closely related to DR incidence. Some studies further adjusted for covariates such as blood lipids, use of glucose-lowering medications, socioeconomic status, education level, physical activity, and history of nephropathy or cardiovascular disease.

The DR outcome ascertainment varied. Most studies employed objective imaging methods such as fundus photography, retinal photography, or direct ophthalmoscopy. Some studies used standardized grading systems for outcome classification, while a few relied on electronic health records, databases, or self-report for outcome identification.

### 3.3 Factors influencing the association between alcohol intake and DR by stage

**Figure 2:**
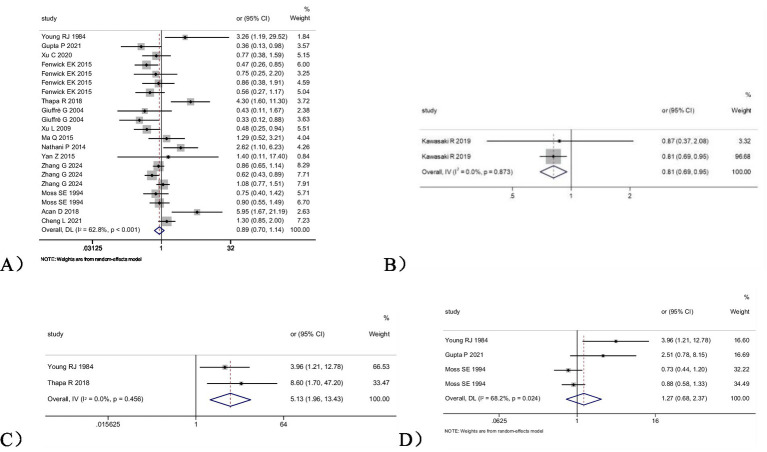
Forest plots of the association between alcohol consumption and risk of diabetic retinopathy, by DR stage. Summary estimates represent odds ratios comparing the highest to lowest intake categories as reported in the studies, with 95% confidence intervals. **(A)** Any DR, random-effects model. **(B)** NPDR, fixed-effect model. **(C)** VTDR, fixed-effect model. **(D)** DR progression, random-effects model. CI, confidence interval; DR, diabetic retinopathy; NPDR, non-proliferative diabetic retinopathy; OR, odds ratio; VTDR, vision-threatening diabetic retinopathy.

Twenty-one studies reported on the association between alcohol intake and overall DR risk, involving 26,139 participants. Due to moderate-to-high heterogeneity (I^2^ = 62.8%), a random-effects model was used for the pooled analysis (Figure 2A). The overall pooled effect estimate was not statistically significant. The funnel plot showed no obvious asymmetry, suggesting no significant publication bias. Egger’s test also indicated no significant publication bias (*p* = 0.570). Sensitivity analysis, performed by omitting one study at a time, showed that the direction and statistical significance of the pooled OR did not change, indicating good robustness of the results. Subgroup analyses stratified by study region, design, and sample size (Supplementary Figures 1C–E) revealed a statistically significant association between alcohol intake and DR risk in the Australian population (OR = 0.59, 95%CI 0.41–0.86, *p* = 0.006), while other subgroups showed no significant associations. This difference might stem from regional population heterogeneity; however, given the risk of false positives due to multiple comparisons in subgroup analyses (37) and the limited number of studies from Australia, this finding requires cautious interpretation and warrants validation in larger, region-specific studies.

One study reported on the association between alcohol intake and non-proliferative diabetic retinopathy (NPDR) risk, involving 5,852 participants. With no heterogeneity detected (*I*^2^ = 0.0%), a fixed-effects model was used (Figure 2B). The pooled effect estimate showed a significant inverse association between alcohol intake and NPDR risk (OR = 0.81, 95%CI 0.69–0.95, *p* = 0.009), suggesting that alcohol intake is inversely associated with NPDR. Internal analysis within this study, stratified by diabetes type (Supplementary Figure 2B), showed a significant inverse association in the type 2 diabetes subgroup (OR = 0.81, 95%CI 0.69–0.95, *p* = 0.010), but not in the type 1 diabetes subgroup, indicating that diabetes type might modify this association.

Two studies reported on the association between alcohol intake and vision-threatening diabetic retinopathy (VTDR) risk, involving 464 participants. With no heterogeneity (*I*^2^ = 0.0%), a fixed-effects model was used (Figure 2C). The pooled effect estimate showed a significant positive association between alcohol intake and VTDR risk (OR = 5.13, 95%CI 1.96–13.43, *p* = 0.001), indicating that alcohol intake is positively associated for VTDR. Subgroup analysis stratified by study design (Supplementary Figure 3B) showed a significant positive association in both cohort studies (OR = 3.96, 95%CI 1.22–12.87, *p* = 0.022) and cross-sectional studies (OR = 8.60, 95%CI 1.63–45.32, *p* = 0.011), with consistent direction of effect across subgroups, further supporting a positive association between alcohol intake and VTDR.

Three studies reported on the association between alcohol intake and overall DR progression risk, involving 1,843 participants. Due to moderate heterogeneity (*I*^2^ = 68.2%), a random-effects model was used (Figure 2D). The overall pooled effect estimate was not statistically significant. Subgroup analysis stratified by sample size showed no statistically significant associations within any subgroup (Supplementary Figure 4B).

### 3.4 Dose-response relationship between alcohol intake and DR by diabetes type

**Figure 3:**
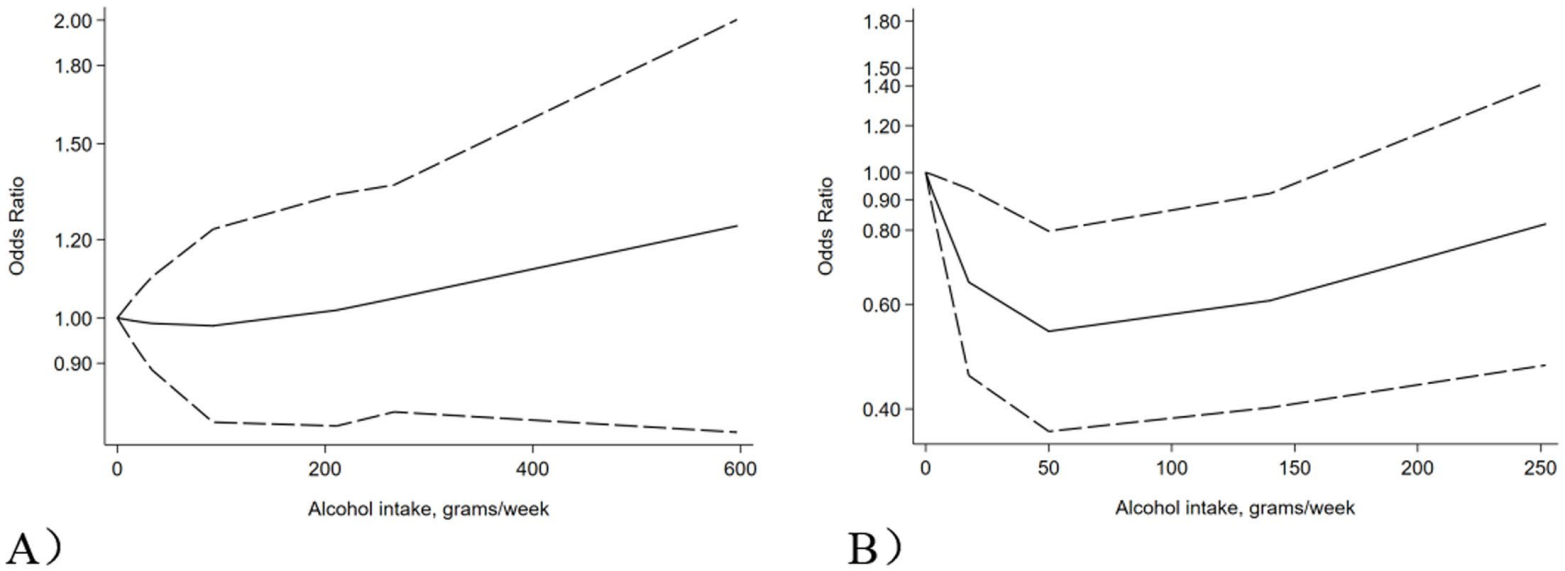
Dose-response relationships between alcohol intake and diabetic retinopathy risk, by diabetes type. Curves were fitted using restricted cubic splines with three knots located at the 10th, 50th, and 90th percentiles of the exposure distribution. Effect estimates are expressed as odds ratios with 95% confidence intervals. **(A)** Type 2 diabetes. **(B)** Type 1 diabetes. CI, confidence interval; DR, diabetic retinopathy; OR, odds ratio.

Six studies reported on the dose-response relationship between alcohol intake and DR risk, involving 11,032 participants. In patients with type 2 diabetes, no significant linear or non-linear dose-response trend was observed between increasing alcohol intake and DR risk. The association between alcohol intake and DR risk was not statistically significant at any dose level (Figure 3A). At an intake of 92 g/week, the effect estimate was 0.98 (95%CI 0.78–1.23); numerical trends suggested a gradual increase in the effect estimate at higher doses (Supplementary Table 4).

In patients with type 1 diabetes, a non-linear dose-response relationship was observed between alcohol intake and DR risk (Figure 3B). From 0 to 50 g/week, the effect estimate decreased continuously with increasing intake, reaching its lowest point at 50 g/week (Exp(xb) = 0.54, 95%CI 0.37–0.80), indicating a significant inverse association of alcohol intake with DR at this dose level. This inverse association remained statistically significant as intake increased from 50 g/week to 140 g/week. At an intake of 252 g/week, the inverse association disappeared (Exp(xb) = 0.82, 95%CI 0.47–1.42) (Supplementary Table 4).

### 3.5 Association between different types of alcoholic beverages and DR

**Figure 4:**
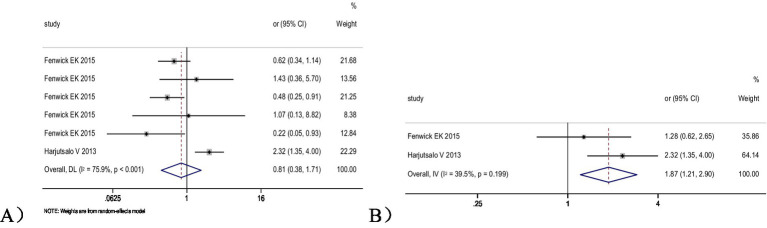
Forest plots of the association between different alcoholic beverage types and diabetic retinopathy. Summary estimates represent odds ratios comparing the highest to lowest intake categories as reported in the studies, with 95% confidence intervals. **(A)** Overall wine consumption, random-effects model. **(B)** Overall spirits consumption, fixed-effect model. Results of subgroup analyses by wine type and spirits by diabetes type are presented in Supplementary Figures 5 and 6. CI, confidence interval; DR, diabetic retinopathy; OR, odds ratio.

Two studies reported on the association between wine consumption and the risk of diabetic retinopathy, involving 4,003 participants. Due to moderate heterogeneity (I^2^ = 75.9%), a random-effects model was used for the pooled analysis (Figure 4A), the overall pooled effect estimate did not reach statistical significance. Subgroup analyses stratified by wine type are detailed in Supplementary Table 6. One independent study reported that white wine (OR = 0.51, 95% CI 0.28–0.95, *p* = 0.035) and sherry (OR = 0.22, 95% CI 0.05–0.95, *p* = 0.042) intake were inversely associated with DR risk (2). In contrast, another independent study reported a positive association between wine intake and DR risk (OR = 2.32, 95% CI 1.35–3.99, *p* = 0.002), which was categorized as “general wine” based on the study’s description (24). The significant heterogeneity in the overall analysis may stem from differences in the definition and classification of wine across studies. This finding suggests potential differences in the biological effects of various wine types; however, given the limited number of included studies and that each wine subtype analysis was based on a single study, these results should be interpreted with caution.

Two studies reported on the association between spirits consumption and the risk of diabetic retinopathy, involving 4,003 participants. With low heterogeneity (*I*^2^ = 39.5%), a fixed-effect model was used for the pooled analysis (Figure 4B). The overall pooled effect estimate showed a significant positive association between spirits consumption and DR risk (OR = 1.87, 95% CI 1.21–2.90, *p* = 0.005). Subgroup analyses stratified by diabetes type are detailed in Supplementary Table 6. A significant positive association was observed in the type 1 diabetes subgroup (OR = 2.32, 95% CI 1.35–3.99, *p* = 0.002), whereas no statistically significant association was found in the type 2 diabetes subgroup. The overall positive association was primarily driven by the significant effect in the type 1 diabetes subgroup.

### 3.6 Association between tea consumption and diabetic retinopathy

**Figure 5:**
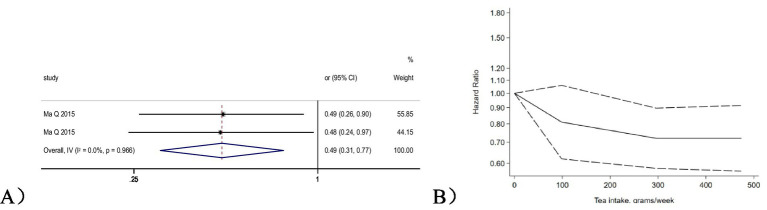
Association between tea consumption and diabetic retinopathy. **(A)** Forest plot of tea consumption and DR. Summary estimate represents odds ratio comparing the highest to lowest intake categories, with 95% confidence interval, fixed-effect model. **(B)** Dose-response relationship between tea consumption (g/week) and DR risk. Curve was fitted using restricted cubic splines. Hazard ratios are shown with 95% confidence intervals. CI, confidence interval; DR, diabetic retinopathy; HR, hazard ratio; OR, odds ratio.

One study reported on the association between tea consumption and DR risk, involving 200 participants. With no heterogeneity (*I*^2^ = 0.0%), a fixed-effects model was used (Figure 5A). The pooled effect estimate showed a significant inverse association (OR = 0.49, 95%CI 0.31–0.77, *p* = 0.002), suggesting an inverse association between tea consumption and DR. Subgroup analysis stratified by current green tea consumption and ever green tea consumption showed statistically significant inverse associations within each subgroup: current (OR = 0.49, 95%CI 0.26–0.91, *p* = 0.024) and ever (OR = 0.48, 95%CI 0.24–0.96, *p* = 0.039), with no significant heterogeneity detected between subgroups.

Another study reported on the dose-response relationship between tea intake and DR risk, involving 6,676 participants. Dose-response analysis revealed a non-linear relationship between tea intake and DR risk (Figure 5B). At an intake of 98.59 g/week, the inverse association was not statistically significant (HR = 0.81, 95%CI 0.62–1.06). At 295.89 g/week, the inverse association became significant (HR = 0.72, 95%CI 0.58–0.90). At 473.39 g/week, the inverse association remained stable and statistically significant (HR = 0.72, 95%CI 0.57–0.91) (Supplementary Table 4). For clinical interpretability, 295 g/week corresponds approximately to 2–3 cups of tea per day (assuming 120 mL per cup).

### 3.7 Association between coffee consumption and diabetic retinopathy

**Figure 6:**
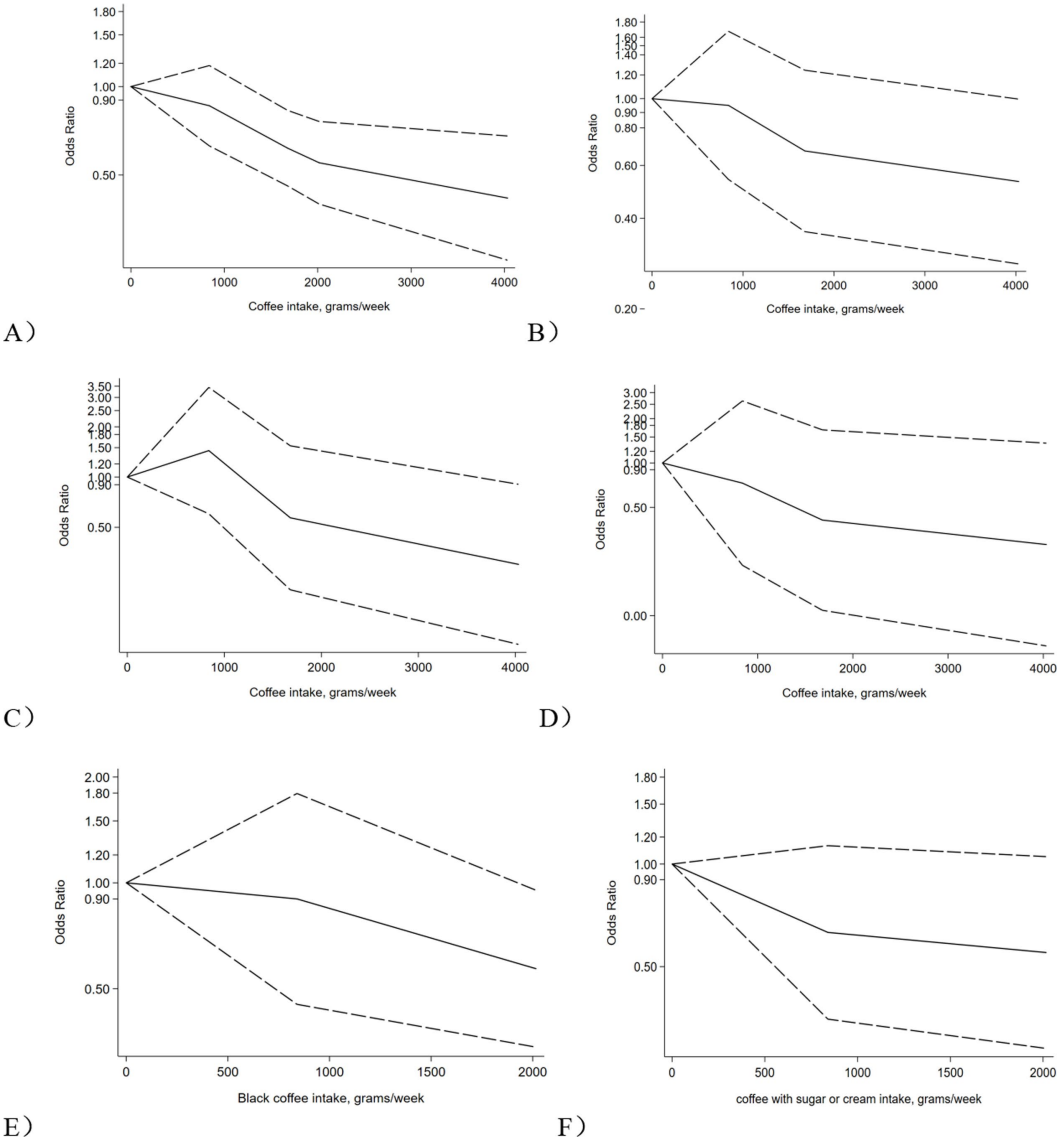
Dose-response relationships between coffee consumption and diabetic retinopathy risk, by DR definition and coffee type. Curves were fitted using restricted cubic splines. Effect estimates are expressed as odds ratios with 95% confidence intervals. **(A)** All studies reporting DR as outcome. **(B)** Any DR. **(C)** VTDR. **(D)** PDR. **(E)** Black coffee. **(F)** Coffee with sugar or cream. CI, confidence interval; DR, diabetic retinopathy; OR, odds ratio; PDR, proliferative diabetic retinopathy; VTDR, vision-threatening diabetic retinopathy.

One study reported on the dose-response relationship between coffee intake and DR risk, involving 1,350 participants. The association between coffee intake and DR exhibited non-linear dose-response characteristics, with results varying by outcome definition. Pooled analysis based on all studies using DR as the outcome (Figure 6A) showed that at intakes of 1,680 g/week (Exp(xb) = 0.62, 95%CI 0.46–0.83) up to 4,032 g/week (Exp(xb) = 0.42, 95%CI 0.26–0.68), the effect estimate showed a continuous decreasing trend, suggesting a more pronounced reduction in DR risk with higher intake. When the analysis was restricted to studies explicitly reporting “any DR” (Figure 6B), the inverse association at high doses became clearer: at 4032 g/week, the pooled effect estimate decreased (Exp(xb) = 0.53, 95%CI 0.28–1.00). At this dose level, coffee was suggestive of a significant inverse association. A similar pattern was observed for vision-threatening diabetic retinopathy (Figure 6C), where at an intake of 4,032 g/week, the effect estimate decreased further (Exp(xb) = 0.30, 95% CI 0.10–0.90), indicating an even more pronounced inverse association. However, the association between coffee intake and the risk of proliferative diabetic retinopathy did not reach statistical significance at any dose level (Figure 6D) (Supplementary Table 4). For clinical interpretability, 4,032 g/week corresponds approximately to 2 cups of coffee per day (assuming 250 mL per cup).

Black coffee consumption showed a non-linear dose-response relationship with diabetic retinopathy (Figure 6E). At an intake of 2016 g/week (Exp(xb) = 0.57, 95% CI 0.34–0.95), black coffee consumption was suggestive of a significant inverse association with DR. Coffee with sugar or cream also showed a non-linear dose-response relationship with diabetic retinopathy (Figure 6F), with no statistically significant associations at any dose level, though the overall trend was toward an inverse association (Supplementary Table 4).

### 3.8 Dose-response relationships between sweetened beverage intake and diabetic retinopathy

**Figure 7:**
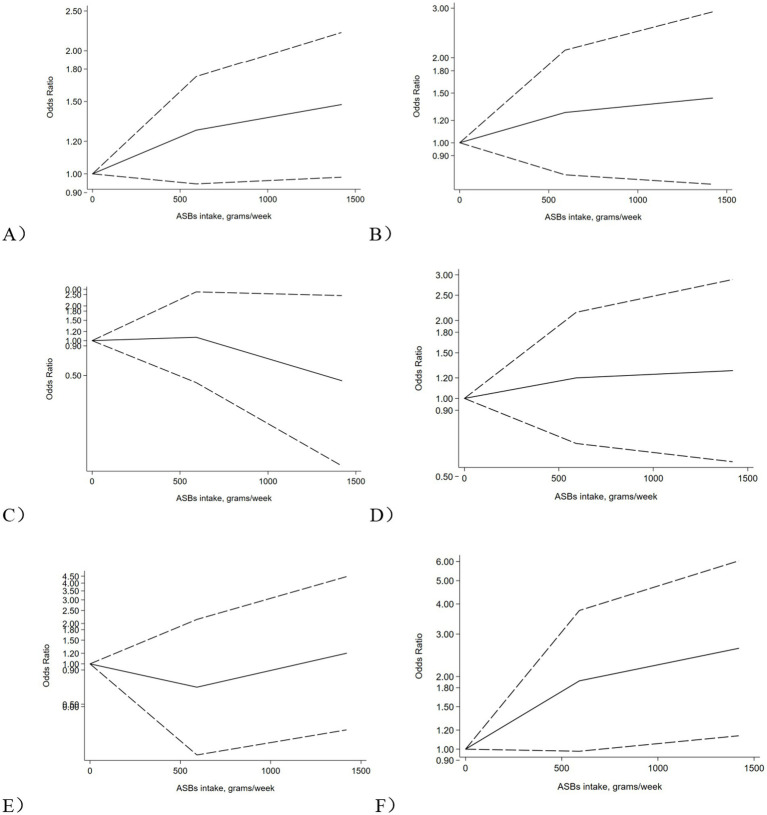
Dose-response relationships between artificially sweetened beverage consumption and diabetic retinopathy risk, by DR severity. Curves were fitted using restricted cubic splines. Effect estimates are expressed as odds ratios with 95% confidence intervals. **(A)** Overall DR, all studies pooled. **(B)** Any DR. **(C)** Mild NPDR. **(D)** Moderate NPDR. **(E)** Severe NPDR. **(F)** PDR. ASB, artificially sweetened beverage; CI, confidence interval; DR, diabetic retinopathy; NPDR, non-proliferative diabetic retinopathy; OR, odds ratio; PDR, proliferative diabetic retinopathy.

One study reported on the association between artificially sweetened beverage (ASB) intake and DR risk, involving 609 participants. ASB intake showed a non-linear dose-response relationship with overall DR occurrence; associations were not statistically significant at any dose level, but the overall trend suggesting a positive association (Figure 7A). ASB intake also showed non-linear dose-response relationships with mild, moderate, and severe NPDR (Figures 7B–E), with no statistically significant associations at any dose level. Importantly, ASB intake showed a non-linear dose-response relationship with PDR (Figure 7F). The effect estimate increased continuously and significantly with increasing intake. At 591.79 g/week, the effect estimate was 1.92, with a 95% confidence interval approaching significance (0.98–3.76). At 1420.2 g/week, the effect estimate increased to 2.62 (95%CI 1.14–6.04), indicating that high-dose ASB intake is significantly associated with higher PDR risk (Supplementary Table 4). For clinical interpretability, 1,420 g/week corresponds approximately to 200 mL per day of artificially sweetened beverages.

**Figure 8:**
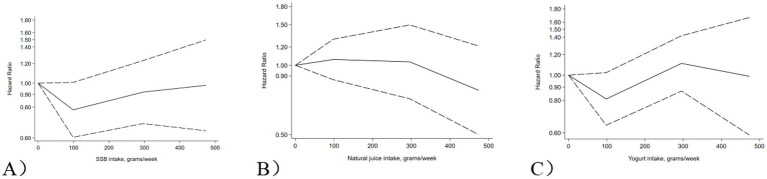
Dose-response relationships between other beverages and diabetic retinopathy risk. Curves were fitted using restricted cubic splines. Effect estimates are expressed as hazard ratios with 95% confidence intervals. **(A)** Sugar-sweetened beverages. **(B)** Natural juice. **(C)** Yogurt. CI, confidence interval; DR, diabetic retinopathy; HR, hazard ratio; SSB, sugar-sweetened beverage.

One study reported on the association between sugar-sweetened beverage (SSB) intake and DR risk, involving 6,676 participants. SSB intake showed a non-linear dose-response relationship with DR risk; associations were not statistically significant at any dose level, with no clear positive or inverse association (Figure 8A and Supplementary Table 4).

One study reported on the association between natural fruit juice intake and DR risk, involving 6,676 participants. Natural fruit juice intake showed a non-linear dose-response relationship with incident DR; associations were not statistically significant at any dose level (Figure 8B). Within the dose range of 0 to 295.89 g/week, the HR showed a slight decreasing trend with increasing intake. At 98.59 g/week, the effect estimate was 1.06 (95%CI 0.87–1.30); at 295.89 g/week, it decreased to 1.03 (95%CI 0.72–1.50); at 473.39 g/week, it further decreased to 0.78 (95%CI 0.50–1.21), suggesting no significant inverse association at this dose level (Supplementary Table 4). Overall, no statistically significant association was found between natural fruit juice intake and incident DR at any dose level. Future large-scale prospective studies are needed to further clarify the impact of different fruit juice types and intake doses on DR risk.

One study reported on the association between yogurt intake and DR risk, involving 6,676 participants. Yogurt intake showed a non-linear dose-response relationship with incident DR; associations were not statistically significant at any dose level, with no clear directional effect (Figure 8C). Within the dose range of 0 to 98.59 g/week, the HR decreased with increasing intake. At 98.59 g/week, the effect estimate was 0.81 (95%CI 0.64–1.02), suggesting no significant inverse association at this dose. At 295.89 g/week, the effect estimate increased to 1.11 (95%CI 0.87–1.42); at 473.39 g/week, it decreased to 0.99 (95%CI 0.59–1.67) (Supplementary Table 4). Overall, no statistically significant association was found between yogurt intake and incident DR at any dose level. However, the current evidence is insufficient; further large-sample prospective studies are needed to assess DR risk associated with yogurt consumption.

## 4 Discussion

### 4.1 Main findings

This systematic review and dose-response meta-analysis comprehensively evaluated the associations between the consumption of nine common beverage types and the risk of diabetic retinopathy. The main findings can be summarized in four key aspects. First, the association between alcohol and DR exhibits significant heterogeneity by disease stage, alcoholic beverage type, and diabetes type-dependent dose-response patterns: no overall association was found, alcohol showed an inverse association with NPDR and a positive association with VTDR; white wine and sherry showed inverse associations, while general wine and spirits showed positive associations; in patients with type 1 diabetes, alcohol intake showed a non-linear inverse association (strongest at 50 g/week, OR = 0.54), whereas no significant dose-response trend was observed in type 2 diabetes. Second, tea consumption was consistently and non-linearly inversely associated with DR, with the inverse association reaching a plateau at approximately 295 g/week (HR = 0.72). Third, the inverse association of coffee was only evident at specific doses and outcome definitions; high doses (4,032 g/week) showed inverse associations with any DR and VTDR, with a clear inverse association for black coffee (at 2016 g/week). Fourth, high-dose intake of artificially sweetened beverages (1,420 g/week) was significantly associated with higher risk of proliferative diabetic retinopathy (OR = 2.62), whereas sugar-sweetened beverages, natural fruit juice, and yogurt showed no significant associations at any dose level.

### 4.2 Heterogeneity and its impact on the interpretation of results

This study did not formally assess the certainty of the body of evidence. Most effect estimates in the current analyses were accompanied by wide confidence intervals, suggesting considerable uncertainty in the estimated effects, and their clinical significance should be interpreted cautiously in conjunction with more robust prospective evidence. Several analyses in this study exhibited moderate to high heterogeneity. Differences across studies in exposure assessment, outcome ascertainment, and population characteristics may all have contributed to this heterogeneity. First, although beverage intake data from the original studies were uniformly converted to g/week for dose-response modeling, the actual cut-off values for the highest and lowest exposure categories still varied across studies. In addition, differences in dietary assessment tools—such as food frequency questionnaires and 24-h dietary recalls—may have introduced varying degrees of measurement error and exposure misclassification, constituting a potential source of heterogeneity. Second, most studies employed objective imaging methods such as fundus photography or direct ophthalmoscopy for DR ascertainment, whereas a few relied on electronic health records or self-report, and such differences in diagnostic methods may have influenced risk estimates. Furthermore, variations in diabetes type, disease duration, geographic region, and genetic background across the included populations may also have affected the associations between beverages and DR. The sets of covariates adjusted for in multivariable models also differed considerably across studies, with some failing to adjust for potential confounders such as physical activity and overall dietary quality; these differences may have contributed to residual confounding and heterogeneity. Although subgroup analyses were conducted to explore potential sources of heterogeneity, the available data were insufficient to fully quantify the contribution of each factor. Therefore, the clinical generalizability of the overall pooled effect estimates should be approached with caution, as the direction and magnitude of the associations between beverages and DR may vary across different populations and healthcare settings.

### 4.3 Comparison with other studies

Previous meta-analyses investigating the association between beverages and DR have several major limitations. The meta-analysis by Zhu et al. published in 2017 included 15 observational studies (12) and found no significant overall association between alcohol intake and DR risk (OR = 0.91, 95%CI 0.79–1.06). Although subgroup analyses suggested potential inverse associations for wine (OR = 0.77) and sherry (OR = 0.22), the analysis remained limited to traditional “highest vs. lowest intake” comparisons, failing to reveal dynamic trends in risk with continuously changing doses. The authors themselves called for future research to focus on the dose and subtypes of alcohol intake. Similarly, the meta-analysis by Chen et al. published in 2020 (3), also including 15 observational studies, reported a null overall association (OR = 0.91, 95%CI 0.78–1.06). While this study noted that the association might be influenced by diabetes type, it similarly lacked dose-response modeling to identify key dose thresholds or non-linear relationships, and the analysis differentiating alcoholic beverage types was insufficient. The authors emphasized the need for future large-sample prospective studies to assess the dose and type of alcohol intake in relation to DR risk.

The present study achieves significant advances over previous work. It is the first to simultaneously include nine beverage categories and apply dose-response modeling, identifying non-linear relationships and critical dose thresholds for tea, coffee, and ASBs with DR risk. Through stage-specific subgroup analyses, it reveals the bidirectional effects of alcohol on different stages of DR. By comparing effect estimates across different alcoholic beverage types, it minimizes between-study confounding. These findings provide a more robust evidence base for refined dietary guidance in patients with DR.

### 4.4 Alcohol intake and diabetic retinopathy

This study systematically reveals that the association between alcohol and DR is characterized by significant stage heterogeneity and type heterogeneity: alcohol intake is significantly inversely associated with NPDR but significantly positively associated with VTDR. Dose-response analysis showed that in patients with type 1 diabetes, alcohol intake exhibited a non-linear inverse association with DR risk: the strongest inverse association was observed at 50 g/week (OR = 0.54, 95%CI 0.37–0.80), persisting within the 50–140 g/week range and disappearing beyond 252 g/week. In contrast, in patients with type 2 diabetes, no significant association was found at any dose level, although numerical trends suggested a gradual increase in effect estimates at intakes ≥92 g/week. These findings explain the null results observed in previous overall analyses and suggest that the impact of alcohol on the retinal microvasculature may change as the disease stage progresses. Studies have shown that ethanol extracts can enhance glucose uptake and insulin sensitivity, exert anti-inflammatory effects, and improve lipid metabolism (38–40), providing a possible biological explanation for the observed results. However, in the vision-threatening stage, ethanol-induced oxidative stress burden, blood pressure fluctuations, and acetaldehyde toxicity may further compromise the already damaged retinal microcirculation (41–43). Subtype analysis of alcoholic beverages further revealed inverse associations for white wine and sherry, a positive association for general wine, and a clear positive association for spirits. It is hypothesized (44, 45) that caffeic acid in white wine and polyphenols in champagne may promote nitric oxide production, mediate vasodilation, and exert anti-inflammatory and antioxidant effects, thereby improving retinal microcirculation perfusion and protecting neurons and endothelial cells from oxidative stress-mediated damage, with associations potentially stronger than those observed for red wine. This hypothesis, however, awaits validation in clinical studies.

### 4.5 Tea consumption and diabetic retinopathy

The findings of this study suggest an inverse association between tea consumption and diabetic retinopathy. Dose-response analysis showed that the inverse association reached a plateau at approximately 295 g/week, and remained stable at higher intake levels. Subgroup analysis revealed no significant difference in the inverse association between former and current tea drinkers, suggesting that regular, long-term tea consumption may be associated with a sustained lower risk of diabetic retinopathy. Studies have demonstrated that tea extracts possess antioxidant, neuroprotective, insulin-sensitizing, and vascular endothelial growth factor–inhibiting properties, which may contribute to neurovascular protection in DR (46–49). However, prospective studies directly investigating the association between tea consumption and diabetic retinopathy remain limited in number and have largely been conducted in Asian populations. This inverse association therefore requires further validation across different geographic regions and diabetes types. The current body of evidence is insufficient to establish a definitive inverse association of tea consumption with DR.

### 4.6 Coffee consumption and diabetic retinopathy

In this study, the inverse association of coffee consumption with diabetic retinopathy was only evident at specific outcome definitions and dose levels. When the analysis was strictly limited to any diabetic retinopathy, an intake of approximately 4,032 g/week was associated with a 47% reduction in risk. The inverse association was even more pronounced for vision-threatening retinopathy, with a 70% risk reduction at the same dose. However, no significant association was found between coffee intake and proliferative retinopathy, and the pooled analysis for overall DR did not reach statistical significance across the entire dose range. These findings suggest that the inverse association of coffee with DR may be primarily concentrated in the moderate-to-severe non-proliferative stages, with limited impact once the disease has progressed to the proliferative stage. The inverse association of black coffee was clear, whereas coffee with sugar or cream showed only a non-significant inverse trend, suggesting that added ingredients may partially offset the potential benefits of coffee. Research indicates that components in coffee, such as chlorogenic acid, caffeine, and trigonelline, possess antioxidant, anti-inflammatory, and anti-angiogenic activities, potentially delaying retinopathy progression by inhibiting hypoxia-inducible factor-1α and vascular endothelial growth factor signaling pathways (50–53). Current epidemiological evidence on coffee and DR remains limited, with dose-response analyses relying on few studies; therefore, conclusions require confirmation in future prospective cohorts. The current epidemiological evidence on coffee and diabetic retinopathy is largely based on dose-response analyses from a single study, with limited sample size and exposure gradients. This is insufficient to conclude that coffee has a definitive inverse association of coffee with DR. Further validation in prospective cohort studies is warranted.

### 4.7 Artificially sweetened beverages and diabetic retinopathy

This study found no significant association between artificially sweetened beverage intake and overall DR or any stage of non-proliferative retinopathy. However, a significant non-linear dose-response relationship was observed specifically with proliferative diabetic retinopathy: at an intake of approximately 1,420 g/week, the risk of PDR was increased 2.62-fold (95%CI 1.14–6.04), representing approximately a 1.6-fold increase in risk. This association was only apparent in the late stage of the disease, suggesting that the positive association of ASBs with DR may be stage-specific. The mechanisms by which artificial sweeteners affect metabolic and vascular health remain controversial. Animal studies suggest (54, 55) that some non-nutritive sweeteners may alter gut microbiota composition, interfere with glucagon-like peptide-1 signaling pathways, and induce endothelial dysfunction, thereby affecting glycemic homeostasis and vascular integrity. However, direct evidence for these mechanisms in humans is insufficient, and results from short-term human intervention trials are inconsistent (56, 57). Currently, population-based studies directly examining the association between artificially sweetened beverages and diabetic retinopathy are extremely scarce. The dose-response analysis in this section is based solely on limited data from a single study; the robustness and reproducibility of this association remain to be confirmed. Furthermore, the possibility that individuals with high intake may themselves have a higher baseline risk, or that reverse causality may be present, cannot be excluded. The conclusions must be interpreted with extreme caution.

### 4.8 Sugar-sweetened beverages, natural fruit juice, yogurt and diabetic retinopathy

This study did not find significant associations between the consumption of sugar-sweetened beverages, natural fruit juice, or yogurt and the risk of diabetic retinopathy. This null finding is not entirely consistent with prior expectations. From a pathophysiological perspective, sugar-sweetened beverages are thought to potentially influence DR risk by inducing acute hyperglycemia, disrupting hepatic metabolism, promoting insulin resistance, and activating oxidative stress and inflammatory pathways (58–60). Although natural fruit juice is rich in vitamins and polyphenolic compounds with glucose-lowering and antioxidant properties (61, 62), its high fructose and sucrose content is thought to potentially offset these benefits through specific metabolic pathways such as increasing uric acid production, activating the polyol pathway, and promoting hepatic *de novo* lipogenesis (58, 59, 63). Yogurt, as a fermented dairy product, contains probiotics, calcium, and bioactive peptides that are thought to improve insulin sensitivity and gut barrier function (64–66); however, free sugars commonly added in commercially available products may attenuate or even reverse such beneficial associations. The mechanistic inferences above are based primarily on animal models and *in vitro* experiments, and direct evidence from long-term human studies with DR endpoints is lacking.

The lack of significant associations for SSBs, natural fruit juice, and yogurt in this study may be attributable to several factors. First, the number of included studies for these three beverages was small, resulting in limited statistical power; dose-response analyses were largely based on single studies, precluding the exclusion of chance findings. Second, substantial heterogeneity existed in exposure assessment methods across studies; some studies did not distinguish between natural fruit juice and sugary fruit drinks, nor between unsweetened and sweetened yogurt products, potentially introducing non-differential misclassification bias. Therefore, the current evidence does not support a direct association between these beverages and DR risk. Regarding SSBs, despite the lack of a statistically significant association in this study, their well-established harms concerning obesity, type 2 diabetes, and cardiometabolic health provide sufficient grounds for clinical recommendations to limit sugar intake.

## 5 Strengths, innovations, and limitations

The finding of no overall association between alcohol and DR in this study aligns with previous meta-analyses; however, it provides a more nuanced analysis through stage-specific subgroup analyses, alcoholic beverage subtyping, and dose-response analysis. Regarding coffee and tea, previous research has largely focused on the risk of type 2 diabetes; this study extends the evidence base to the field of diabetic retinopathy and reports, for the first time, inverse dose-response relationships. The significant association between artificially sweetened beverages and proliferative diabetic retinopathy has only been reported sporadically in prior literature.

The strengths of this study include being the first to simultaneously cover nine beverage categories, integrate dose-response analysis, strictly adhere to PRISMA and MOOSE guidelines, and the overall high quality of the included studies. The application of dose-response meta-analysis allowed us to overcome the limitations of traditional “highest vs. lowest intake” comparisons, revealing non-linear relationships and dose thresholds for beverages like tea, coffee, and ASBs in relation to DR risk, providing an evidence base for refined dietary guidance.

The evidence synthesized in this study was derived entirely from observational studies. In the field of diet and chronic disease, residual confounding and reverse causality are well-recognized obstacles to causal inference from observational data (67). Cross-sectional studies accounted for a substantial proportion of the included evidence, precluding establishment of the temporal sequence between exposure and outcome. Patients diagnosed with DR may subsequently alter their dietary behaviors, making reverse causality an important concern. Most studies relied on self-reported questionnaires for beverage intake data, and recall bias is difficult to avoid. Although the majority of studies adjusted for key variables such as age, HbA1c, and diabetes duration, the sets of adjusted covariates varied considerably across studies. Data from NHANES 2011–2016 showed that adult tea consumers had significantly better dietary quality than non-consumers (68); similarly, the UK Biobank cohort observed healthier overall dietary patterns among coffee and tea drinkers (69). In contrast, consumers of artificially sweetened beverages had poorer dietary quality than non-consumers (70). Such lifestyle differences associated with beverage choice are difficult to fully measure and adjust for. In addition, considerable heterogeneity existed across studies in beverage intake assessment tools and DR stage definitions. Therefore, “protective effects” or “harmful effects” described throughout the text should be interpreted as statistical associations rather than causal inferences.

Methodological limitations of this review primarily include the following: considerable heterogeneity across studies in beverage intake measurement, DR stage definitions, and confounder adjustment; limited numbers of studies for some beverages, with dose-response analyses based on few or single studies, requiring cautious interpretation of the findings; subgroup analyses based on small numbers of studies, without formal interaction tests or adjustment for multiple comparisons, which carries a risk of false-positive findings.

A structured GRADE assessment was performed for the main analyses, and the ratings are presented in Supplementary Table 7. Most associations were rated as providing low or very low certainty evidence. The dose-response relationships for alcohol in type 1 diabetes and for tea were rated as moderate certainty owing to the presence of a dose-response gradient. It should be noted, however, that the application of the GRADE framework to systematic reviews of observational studies has inherent limitations. The starting rating for observational evidence is “low certainty” by default, and upgrading criteria—such as a dose-response gradient—are intended primarily for studies of potential harm from environmental or occupational exposures rather than studies of potentially beneficial dietary exposures. Moreover, factors such as heterogeneity in exposure measurement tools and broad confidence intervals, both of which were commonly encountered in the included studies, introduce additional uncertainty that may not be fully captured by the GRADE criteria. The ratings presented here should therefore be interpreted as a structured, transparent summary of the current evidence rather than a definitive judgment of the quality of the underlying studies.

## 6 Research recommendations and clinical implications

For future research, there is an urgent need for large-scale, prospective, multi-center cohort studies employing standardized beverage frequency questionnaires and retinal imaging grading. Beverage types should be finely distinguished. Proliferative diabetic retinopathy should be considered a core outcome in studies of artificially sweetened beverages. Metabolomics and mediation analyses should be conducted to elucidate the molecular pathways through which alcohol, tea polyphenols, and caffeine influence DR.

For clinical practice, it is not recommended that non-drinking diabetic patients initiate alcohol consumption. For current drinkers, the increased risk of DR associated with spirits should be emphasized. If wine is preferred, white wine or sherry may be preferable, with total intake controlled. Moderate, long-term tea consumption (approximately 2–3 cups daily) may be beneficial for diabetic patients. Coffee need not be avoided, as high doses may be associated with a lower risk of DR; however, caution regarding the adverse effects of added sugar and creamers is warranted. Artificially sweetened beverages should be consumed with caution, especially in high doses, by patients with existing retinopathy. For SSBs, natural fruit juice, and yogurt, while no specific DR evidence was found, dietary guidelines for diabetes recommending limiting free sugar intake and prioritizing water or unsweetened tea as primary beverages should still be followed. These recommendations should be carefully weighed in the context of evidence quality and certainty, and tailored to individual patient circumstances.

## 7 Conclusion

This systematic review and dose-response meta-analysis demonstrates that the association between alcohol and diabetic retinopathy exhibits dual characteristics of stage dependence and type dependence: alcohol intake is inversely associated with NPDR risk and positively associated with VTDR risk; white wine and sherry intake are inversely associated with DR risk, while spirits intake is positively associated with DR risk. Tea and high-dose coffee consumption are inversely associated with DR risk, displaying non-linear trends; high-dose intake of artificially sweetened beverages is positively associated with PDR risk. Current evidence is insufficient to support independent associations of sugar-sweetened beverages, natural fruit juice, or yogurt with DR. These findings are derived from observational studies and are limited by the high proportion of cross-sectional studies, the reliance of several analyses on single studies, and methodological heterogeneity across studies. Most conclusions therefore require validation in larger, prospective studies. Future research should prioritize prospective investigations of artificially sweetened beverages, tea, and coffee, report outcomes at comparable DR stages, and adopt standardized beverage intake assessment tools.

## Data Availability

The original contributions presented in the study are included in the article/Supplementary material, further inquiries can be directed to the corresponding authors.
