# Supplementary material for: Differential associations of beverage consumption with diabetic retinopathy: a systematic review and dose-response meta-analysis of observational studies
Source: Front Med (Lausanne). 2026 Jun 17;13:1819832. doi: 10.3389/fmed.2026.1819832 (PMC13318734; doi:10.3389/fmed.2026.1819832)
Supplement: Supplementary file 1 [file Data_Sheet_1.PDF]

Supplementary Table 1 Literature Search Strategy for PUBMED, EMBASE, Web of Science and COCHRANE Databases

PUBMED	((((((((((((((((((beverage[Title/Abstract]) OR (tea[Title/Abstract])) OR (alcohol[Title/Abstract])) OR (wine[Title/Abstract])) OR (beer[Title/Abstract])) OR (liquor[Title/Abstract])) OR (coffee[Title/Abstract])) OR (soda[Title/Abstract])) OR (soft drinks[Title/Abstract])) OR (diet[Title/Abstract])) OR (carbonated beverage[Title/Abstract])) OR (soda pop[Title/Abstract])) OR (cola beverage[Title/Abstract])) OR (cola drink[Title/Abstract])) OR (cordial[Title/Abstract])) OR (cordial beverage[Title/Abstract])) OR (cordial drink[Title/Abstract])) OR (flavoured water[Title/Abstract])) OR (artificial juices[Title/Abstract]))) AND ((((((Diabetic retinopathy[Title/Abstract]) OR (diabetic retinopathy[MeSH Terms])) OR (diabetic retinal disease[Title/Abstract])) OR (diabetic macular oedema[Title/Abstract])) OR (DMO[Title/Abstract])) OR (DME[Title/Abstract]))
EMBASE	'beverage':ab,kw,ti OR 'tea':ab,kw,ti OR 'alcohol':ab,kw,ti OR 'wine':ab,kw,ti OR 'beer':ab,kw,ti OR 'liquor':ab,kw,ti OR 'coffee':ab,kw,ti OR 'soda':ab,kw,ti OR 'soft drinks':ab,kw,ti OR 'diet':ab,kw,ti OR 'carbonated beverage':ab,kw,ti OR 'soda pop':ab,kw,ti OR 'cola beverage':ab,kw,ti OR 'cola drink':ab,kw,ti OR 'cordial':ab,kw,ti OR 'cordial beverage':ab,kw,ti OR 'cordial drink':ab,kw,ti OR 'flavoured water':ab,kw,ti OR 'artificial juices':ab,kw,ti AND 'Diabetic retinopathy':ab,kw,ti OR 'diabetic retinal disease':ab,kw,ti OR 'diabetic macular oedema':ab,kw,ti OR 'DMO':ab,kw,ti OR 'DME':ab,kw,ti
WEB OF SCIENCE	(TS=(beverage OR tea OR alcohol OR wine OR beer OR liquor OR coffee OR soda OR soft drinks OR diet OR carbonated beverage OR soda pop OR cola beverage OR cola drink OR cordial OR cordial beverage OR cordial drink OR flavoured water OR artificial juices)) AND TS=(Diabetic retinopathy OR diabetic retinal disease OR diabetic macular oedema OR DMO OR DME)
COCRANE	(beverage):ti,ab,kw OR (tea):ti,ab,kw OR (alcohol):ti,ab,kw OR (wine):ti,ab,kw OR (beer):ti,ab,kw OR (liquor):ti,ab,kw OR (coffee):ti,ab,kw OR (soda):ti,ab,kw OR (soft drinks):ti,ab,kw OR (diet):ti,ab,kw OR (carbonated beverage):ti,ab,kw OR (soda pop):ti,ab,kw OR (cola beverage):ti,ab,kw OR (cola drink):ti,ab,kw OR (cordial):ti,ab,kw OR (cordial beverage):ti,ab,kw OR (cordial drink):ti,ab,kw OR (flavoured water):ti,ab,kw OR (artificial juices):ti,ab,kw AND MeSH descriptor: [Diabetic Retinopathy] explode all trees OR (diabetic retinopathy):ti,ab,kw OR (diabetic retinal disease):ti,ab,kw OR (diabetic macular oedema):ti,ab,kw OR (DMO):ti,ab,kw OR (DME):ti,ab,kw

Supplementary Table 2 Study characteristics

Author	Publication year	Study design	Country	Sample size	Percent of male (%)	Mean age (year)	Follow -up (years)	Number of cases	Exposure	Exposur measurement	DR diagnosis	DR definition	Diabetes	Exposure Definition	Adjustment/matched	NOS score
Young RJ	1984	Cohort	UK	296	100.0	20.0-59.0	4.7	66	Alcohol	Questionnaire	Direct ophthalmoscopy	Four Grades	Mixed	⩽10 measures/ week,>10 measures/week	Crude	7
Beulens JWJ	2008	Cross-sectional	UK	3,250	29.7	40.1	7	304	Alcohol	Questionnaire	Retinal photography	Proliferative retinopathy	T1DM	0 g/week, 0.0 – 4.9 g/week, 5.0 – 29.9 g/week, 30.0 – 69.9 g/week, 70.0 – 209.9 g/week, ⩾210 g/week	Age, sex, centre, duration of diabetes, systolic BP, physical activity, smoking, BMI, HbA1c, cardiovascular disease	8
Lee CC	2010	Cohort	UK	1239	60.7	65	5.5	182 (ETDRS 2-step progression) 640 (Any retinal vascular lesions)	Alcohol	Self-reported questionnaire	Stereoscopic seven-field retinal photography	1.2-step progression in ETDRS score 2. Presence of any retinal vascular lesions	T2DM	0 drinks/week, 1 – 14 drinks/week, >14 drinks/week	Age, sex, HbA1c, systolic BP, diabetes duration, BMI, cigarette smoking, ethnicity, treatment interventions	8
Gupta P	2020	Cohort	Singapore	656	54.4	58.8	6	82 (Incident DR) 45 (DR Progression)	Alcohol	Self-reported questionnaire	Two-field fundus photography	1. Incident DR: No DR at baseline → ⩾ minimal NPDR at follow-up. 2. DR Progression: ⩾1-step worsening on Modified Airlie House scale (excluding PDR).	T2DM	1.Overall: Abstainers,Consumers. 2. Frequency: Abstainers, Infrequent (⩽2 days/week) ,Frequent (>2 days/week)	Model 2: Age, sex, BMI, current smoking, SBP, income, HbA1c, diabetes duration, hyperlipidemia, CKD, antidiabetic medication use, presence of other eye conditions	8
Lin X	2025	Cohort	China	6676	58.0	60.1	11.7	548	Artificially Sweetened Beverages (ASBs), Sugar-Sweetened Beverages (SSBs), Tea, Coffee, Natural Juice, Yogurt	Oxford WebQ online 24-hour dietary recall	Hospital inpatient admissions & death registries	DR	T2DM	Categorical: e.g., for ASBs/SSBs/Tea: 0, 0.1-0.9, 1.0-1.9, ⩾2.0 units/day. Substitution analysis: Replacing 0.5 unit/day of ASBs/SSBs with other beverages.	Age, sex, Townsend Deprivation Index, ethnicity, education, BMI, smoking, drinking status, physical activity, healthy diet score, total energy, family history of CVD, hypertension, eGFR, aspirin use, lipid-lowering & diabetes medication use, diabetes duration, HbA1c.	9
Lee HJ	2022	Cross-sectional	Korea	1350	55.5	58.5	Not Applicable	270	Coffee	Food frequency questionnaire; 24-h dietary recall	Fundus photography	Modified Airlie House classification	T2DM	Almost none; <1 cup/day; 1 cup/day; ≥2	Age, sex, education, occupation, income, smoking, alcohol, BMI,	9

Author	Publication year	Study design	Country	Sample size	Percent of male (%)	Mean age (year)	Follow-up (years)	Number of cases	Exposure	Exposur measurement	DR diagnosis	DR definition	Diabetes	Exposure Definition	Adjustment/matched	NOS score
														8cups/day	physical activity, hypertension, dyslipidemia, diabetes duration, HbA1c, energy intake	
Fenwick EK	2018	Cross-sectional	Australia	609	34.5	64.6	Not Applicable	Any DR: 379; PDR: 146	Diet soft drink; Regular soft drink	Food frequency questionnaire	Fundus photography; OCT	ETDRS scale (Levels: 10-15, 20, 31-43, 53-60, 61-80); AAO scale for DME	Mixed (T1DM & T2DM)	D9iet soft drink: No (<1 can/week), Moderate (1-4 cans/week), High (>4 cans/week). Regular soft drink: No (<1 can/week), Any consumption (≥1 can/week).	Age, gender, HbA1c, SBP, diabetes duration, insulin use, presence of ≥1 other diabetes complication, diabetes type, BMI, education, antihypertensive medication, hyperlipidemia, comorbidity, smoking, alcohol, total energy intake, and (for diet) regular soft drink consumption.	8
Kawasaki R	2019	Cross-sectional	Japan	5852	58.7	61.1	Not Applicable	NPDR total: 1598 (T1DM: 83; T2DM: 1515)	Alcohol	Questionnaires	Fundus photography	International clinical DR severity scale	Mixed (T1DM & T2DM	Alcohol,No Alcohol	Model adjusted for age, sex, HbA1c, diabetes duration, BMI, blood pressure, lipids, eGFR, smoking, medication use, etc.	7
Yamamoto M	2025	Cohort	Japan	21,392	100.0	52 - 53	4.3	425	Alcohol	Baseline questionnaire	Claims database (ICD-10 codes) & medical procedures	Treatment-required diabetic eye disease	T2DM	Quantity per occasion (QPO): <1 drink, ⩾1 to <2 drinks, ⩾2 to <3 drinks, ⩾3 drinks. Frequency per week (FAC): None or ⩽1/wk, 2-4/wk, ⩾5/wk. Total weekly intake: <100g, 100-300g, >300g ethanol.	Age, BMI, HbA1c, fasting glucose, SBP, LDL-C, HDL-C, triglycerides, proteinuria, medication use, smoking.	9
Xu C	2020	Cross-sectional	China	5281	47.83	67.90	N/A	54	Tea consumption	Face-to-face questionnaire interview	Retinal fundus imaging	Levels 14–85	Fasting glucose ≥7.0 mmol/L or self-reported diagnosis	Age, gender, occupation, education, income, smoking, alcohol, duration of diabetes, BMI, FBG, TG, HDL-C, SBP	Not applicable (cross-sectional)	9
Fenwick EK	2015	Cross-sectional	Australia	395	64.1	65.9	N/A	235 (any DR)	Alcohol consumption	Self-administered FFQ	Two-field fundus photography	Non-VTDR: mild/moderate NPDR; VTDR:	T2DM	Abstainers: <1 std drink/week; Moderate: 1–14	Age, gender, smoking, BMI, systolic BP, diabetes control	8

Author	Publication year	Study design	Country	Sample size	Percent of male (%)	Mean age (year)	Follow-up (years)	Number of cases	Exposure	Exposur measurement	DR diagnosis	DR definition	Diabetes	Exposure Definition	Adjustment/matched	NOS score
												severe NPDR, PDR, and/or severe macular edema		std drinks/week; High: >14 std drinks/week	(HbA1c ≥7%), insulin use, diabetes duration, presence of at least one other diabetes complication	
li B	2024	Cross-sectional	China	3482	51.2	52.2	N/A	767	Alcohol consumption	Self-reported frequency and quantity	Self-reported diagnosis by a doctor	Self-reported diabetic retinopathy	Self-reported diagnosis by a physician	No drinking, Mild to moderate (<1 drink/day women, <2 men), Heavy (≥1 drink/day women, ≥2 men)	Age, sex, race/ethnicity, diabetic duration, HbA1c control, blood pressure control, lipid control (non-HDL), diabetic nephropathy, cardiovascular diseases	5
Harjutsalo V	2013	Cross-sectional	Finland	3,608	Not explicitly given in extracted text	37.4	N/A	1,191	Alcohol consumption	Questionnaire	Severe diabetic retinopathy	History of laser photocoagulation for severe retinopathy	T1DM	Lifelong abstainers (0 g/week), Light consumers (Men: 0–83.9 g/week; Women: 0–59.9 g/week), Moderate (Men: 84–287.9; Women: 60–191.9), Heavy (Men: ≥288 g/week; Women: ≥192 g/week); Beverage type: >75% of consumption as wine, beer, or spirits	Age at onset of diabetes, sex, duration of diabetes, triglycerides, HDL cholesterol, HbA1c, social class, BMI, smoking status, hypertension, lipid-lowering medication	8
Thapa R	2018	Cross-sectional		168	43.5	67.9	N/A	40	Alcohol consumption	Self-reported	Clinical examination	Any DR (mild NPDR, moderate NPDR, severe NPDR, PDR); Vision-threatening DR	Diabetes	Alcohol consumers (present or past) , no alcohol consumption (amount not recorded)	Multivariable: duration of diabetes, systolic BP	9
Giuffre G	2004	Cross-sectional		1019	44.9	Not reported	Not applicable	45	Alcohol	Questionnaire	Ophthalmoscopy, fundus photos, fluorescein angiography	Nonproliferative and proliferative DR	Self-reported diabetes	Duration of alcohol consumption	Age, sex, diabetes duration, treatment type	6
Xu L	2009	Population-based cross-sectional study	China	4141	43.4%	55.8	N/A	366	Alcohol	Self-reported questionnaire	Fundus photographs	NA	Mixed	Consumers, Non-consumers	Multivariate: Age, gender, rural/urban region, level of education, smoking , Univariate comparisons also made for systemic/ocular parameters	9

Author	Publication year	Study design	Country	Sample size	Percent of male (%)	Mean age (year)	Follow -up (years)	Number of cases	Exposure	Exposur measurement	DR diagnosis	DR definition	Diabetes	Exposure Definition	Adjustment/matched	NOS score
Ma Q	2015	Clinic-based case-control	China	200	34.0% (68/200)	64.8	N/A	100	Chinese green tea	Face-to-face interview / questionnaire	Retinal photographs , clinical exam	ETDRS classification	T2DM	Ever regularly drunk every week for ≥1 year , Never	Multivariate: Age, sex, education, BMI, systolic BP, smoking, alcohol, diabetes duration, insulin therapy, family history of diabetes, physical activity, fasting glucose. Design: Age- and sex-matched controls.	8
Martin-Merino E	2017	Nested case-control within a cohort	United Kingdom	2,405	~57%	64	N/A	211	Alcohol	Electronic health records	Computerized records (Read codes), validated via manual review and PCP questionnaires	Clinical diagnosis of Diabetic Macular Oedema	T2DM	High alcohol use	Multivariate logistic regression: Adjusted for sex, age, time since diabetes onset, PCP visits, and other significant factors	9
Nathani P	2024	Matched case-control	India	252	Not explicitly stated	63.5	N/A	126	Alcohol	Questionnaire	Clinical exam	ETDRS protocol	Mixed	Alcoholic or Ex-alcoholic, Non-drinker	Multivariate logistic regression: Adjusted for insulin, smoking, medication irregularity, alcohol, sedentary lifestyle, dietary nonadherence, other microvascular complications, dyslipidemia, hypertension. Design: Age- (±2 years) and sex-matched controls.	5
Yan Z	2016	Retrospective cross-sectional	China	1100	43.9	~52.9	Not applicable	307	Alcohol consumption	Self-reported (≥1 drink/week ,<1 drink/week)	Retinal photography, graded using ETDRS severity scale	Presence of ≥1 definite microaneurysm or other characteristic lesions	T2DM	Drinkers: ≥1 drink/week; Non-drinkers: <1 drink/week	Age, gender	6
Martin-Merino E	2016	Nested case-control	UK	17130	Cases: 57.6%, Controls: 54.5% (approx. Over all: ~56%)	Cases: 63 (SD 11.7) at DR diagnosis	Not specified	7735	Alcohol consumption	Self-reported (units/week; 1 unit = 10mL/8g ethanol). Most recent status before diabetes onset.	READ codes in primary care records . Validation: manual review, GP questionnaire	Incident diagnosis of retinopathy related to diabetes (READ codes). Background/non-proliferative: 52.6%, Proliferative: 1.4%, Unspecified: 46%	Incident Type 2 diabetes	Categories (units/week): 0-1, 2-21, 22-34, ≥35. Exposed (primary analysis): High consumption (≥35 units/week).	Fully adjusted model: sex; age at index date; diabetes duration; PCP visits; referrals & hospitalizations; smoking; alcohol consumption; first HbA1c; systolic BP; glaucoma; cataracts/lens extraction; HDL &	9

Author	Publication year	Study design	Country	Sample size	Percent of male (%)	Mean age (year)	Follow -up (years)	Number of cases	Exposure	Exposur measurement	DR diagnosis	DR definition	Diabetes	Exposure Definition	Adjustment/matched	NOS score
															triglycerides; hypoglycaemic agents.	
Zhang G	2024	Prospective cross-sectional	China	7274	43%	Not explicitly stated in provided text for the total sample.	Not applicable	Any DR: 3054 DME: 1153 STDR: 1500	Alcohol consumption	Self-reported frequency	Color fundus photography (2 fields) and Spectral Domain Optical Coherence Tomography (SD-OCT). Graded by trained senior graders/ophthalmologists.	Based on revised International Diabetic Retinopathy Classification Criteria. DR: R1, R2, R2.5, R3. DME: OCT criteria (retinal thickening, cysts, fluid) or fundus photo criteria (exudates near fovea). STDR: R2.5, R3, or any DR grade with maculopathy (M1).	Mixed	Categories (Frequency): Never ; Less than once a week; At least once a week; Every day. Protective effect reported for: Drinking at least once a week.	Multivariate logistic regression adjusting for age and sex (initial model), then further adjusted for other significant factors (BMI, duration, lab values, etc.) as shown in Table 2.	7
Moss SE	1994	Prospective cohort	Not explicitly stated in excerpt	Baseline (1984-86): Younger-onset: 891; Older-onset: 987. Analysis for progression: Younger-onset: 439; Older-onset: 478. Analysis for incidence: Younger-onset: 46; Older-onset: 193.	Not explicitly stated in excerpt.	Not explicitly provided as a mean.	6	Incidence, Progression, Progression to PDR rates are provided in Table 3 (see PDF), but raw case numbers are not explicitly stated in the excerpt.	Alcohol consumption	Questionnaire (self-reported). Summarized as average or recent consumption	Stereoscopic color fundus photographs, graded by standard protocols.	Severity graded from photographs. Outcomes: Incidence of retinopathy, Progression of retinopathy, Progression to proliferative retinopathy.	Younger-onset: Diagnosis before age 30 and taking insulin (likely Type 1). Older-onset: Diagnosis after age 30.	Categories (for trend analysis): Nondrinkers, Light, Moderate, Heavier drinkers. Quantitative (for OR): Increase per 1 oz/day (≈29.6 mL/day). Proportion heavier drinkers: ~6% younger-onset, ~4% older-onset.	Multivariate logistic regression models adjusted for: Model 1/2 (Younger): Age, Sex, Glycosylated hemoglobin, Retinopathy severity (for progression). Model 1/2 (Older): Age, Duration of diabetes, Glycosylated hemoglobin, Retinopathy severity (for progression).	9
Acan D	2018	Cross-sectional	Turkey	413	Overall: 46.2 % male (191/413). DME group: 58.7% male (37/63). Non-DME	Overall: ~56.5 (calculated from groups). DME group: 5	Not applicable	DME cases: 63 (15.3%) DR cases: 132 (32%)	Alcohol consumption	Patient history/habits	Dilated fundoscopy, Fluorescein Angiography (FA), Optical Coherence Tomography	DME: Central Macular Thickness (CMT) via OCT ≥ 250 μm attributable to DME and/or Clinically	Type 1 and Type 2 Diabetes (diagnosed by an endocrinologist).	Binary (in analysis): Alcohol consumers vs. Non-consumers. Prevalence: 10/413 (2.4%) were alcohol consumers.	Univariate analysis (Chi-squared, t-test). No multivariate regression model specifically for alcohol was detailed in the provided excerpts.	7

Author	Publication year	Study design	Country	Sample size	Percent of male (%)	Mean age (year)	Follow -up (years)	Number of cases	Exposure	Exposur measurement	DR diagnosis	DR definition	Diabetes	Exposure Definition	Adjustment/matched	NOS score
					group: 44.0% male (154/350).	8.86 ± 11.27. Non-DME group: 56.03 ± 11.95.		PDR cases: 46 (11.1% of total, 34.8% of DR cases)				Significant Macular Edema (CSME) by ETDRS criteria. DR severity: Mild-moderate NPDR, Severe NPDR, Proliferative DR (PDR). Grading based on clinical examination/FA.	T1DM: 27 (6.5%) T2DM: 386 (93.5%)	Association: 5/63 (7.9%) DME patients were consumers vs. 5/350 (1.4%) non-DME patients (p=0.010).	Adjustment was not performed in a combined model; factors were analyzed separately.	
Cheng L	2021	Cross-sectional	China	4053	36.0	51.89 ± 8.94	Not applicable	Total DR cases (ETDRS≥20): 163 NGR group: 53 IGR group: 52 Newly diagnosed T2DM group: 58	Alcohol consumption history	Questionnaire	Non-mydriatic digital fundus photography (Canon CR-2AF), two fields per eye (optic disc and macula). Centralized grading by two professional ophthalmologists (blinded).	Early Treatment Diabetic Retinopathy Study (ETDRS) grading. DR defined as: ETDRS severity level ≥20 ("diabetes-specific" retinopathy). Severity: Mild NPDR, Moderate NPDR, Severe NPDR.	Participants stratified by glucose metabolism status: Normal Glucose Regulation (NGR): FPG <6.1 mmol/L AND 2hPG <7.8 mmol/L. Impaired Glucose Regulation (IGR): Pre-diabetes. Newly diagnosed Type 2 Diabetes: FPG ≥7.0 mmol/L and/or 2hPG ≥11.1 mmol/L. (Excluded previously diagnosed diabetics).	Alcohol consumption history: Defined as alcohol intake > 30 grams per week for more than one year.	1:4 matched case-control analysis. Cases: 163 DR patients. Controls: 812 matched by sex and age (±5 years). Multivariate model: Conditional logistic regression adjusting for factors in the model (SBP categories, BMI, Waist-to-Height Ratio, FPG).	10

Supplemental Table 3. Details of quality assessment of included studies in the systematic review and meta-analysis based on Newcastle-Ottawa Scale[1].

A.Cohort and nested case-control studies.

	SELECTION				COMPARABILITY	OUTCOME			
	Representativeness of the exposed cohort	Selection of the non-exposed cohort	Ascertainment of exposure	Demonstration that outcome of interest was not present at start of study	Comparability of cohorts on the basis of the design or analysis	Assessment of outcome	Was follow-up long enough (5year)for outcomes to occur	Adequacy of follow up of cohorts	Total score
Young RJ et al, 1984	*	*	*	*	**	*			7
Lee CC et al, 2010	*	*	*	*	**	*	*		8
Gupta P et al, 2020	*	*	*	*	**	*	*		8
Lin X et al, 2025	*	*	*	*	**	*	*	*	9
Kawasaki R et al, 2019	*	*	*		**	**			7
Yamamoto M et al, 2025	*	*		*	**	*	*	**	9
Giuffrè G et al, 2004	*	*	*	*	*	*			6
Martín-Merino E et al, 2017	*	*	*	*	**	*	*	*	9
Martín-Merino E et al, 2016	*	*	*	*	**	*	*	*	9
Moss SE et al, 1994	*	*	*	*	**	*	*	*	9

B. Cross-sectional studies.

	SELECTION				COMPARABILITY	OUTCOME		
	Representativeness of the sample	Sample size	Non-respondents	Ascertainment of the exposure (risk factor)	Comparability of subjects in different outcome groups	Assessment of outcome	Statistical test	Total score
Beulens JWJ et al, 2008	*	*	*	**	**	*	*	8
Lee HJ et al, 2022	*	*		**	**	**	*	9
Fenwick EK et al, 2018		*	*	*	**	**	*	8
Xu C et al, 2020	*	*	*	*	**	**	*	9
Fenwick EK et al, 2015		*	*	*	**	**	*	8
Li B et al, 2024	*	*	*		*		*	5
Harjutsalo V et al, 2013	*	*		*	**	**	*	8
Thapa R et al, 2018	*	*		**	**	**	*	9
Giuffrè G et al, 2004	*	*	*	*	**	**	*	9
Xu L et al, 2009	*	*	*	*	**	**	*	9
Yan Z et al, 2016		*		*	*	**	*	6
Zhang G et al, 2024		*		**	*	**	*	7
Acan D et al, 2018		*		**	*	**	*	7
Cheng L et al, 2021	*	*	*	**	**	**	*	10

C. Case-Control Studies

	SELECTION				COMPARABILITY	OUTCOME			
	Is the case definition adequate?	Representativeness of the cases	Selection of Controls	Definition of Controls	Comparability of cases and controls on the basis of the design or analysis	Ascertainment of exposure	Same method of ascertainment for cases and controls	Non-Response rate	Total score
Ma Q et al, 2015	*	*	*	*	**	*	*		8
Nathani P et al, 2024	*		*	*	*		*		5

Supplementary Table 4 Dose-response meta-analysis of beverage consumption and diabetic retinopathy: summary of exposure-outcome associations from included studies

Table 1. Dose-response relationship between alcohol consumption and diabetic retinopathy in patients with type 2 diabetes.

Dose	Exp(xb)	L95%CI	U95%CI
0	1.00	1.00	1.00
4	1.00	0.98	1.01
5.25	1.00	0.98	1.02
14	0.99	0.95	1.04
25.200001	0.99	0.91	1.08
33.599998	0.99	0.89	1.10
92	0.98	0.78	1.23
211.2	1.02	0.78	1.33
266.29999	1.05	0.80	1.36
596.5	1.24	0.77	2.00

Table 2. Dose-response relationship between alcohol consumption and diabetic retinopathy in patients with type 1 diabetes.

Dose	Exp(xb)	L95%CI	U95%CI
0	1.00	1.00	1.00
2.5	0.93	0.88	0.99
17.5	0.65	0.46	0.94
50	0.54	0.37	0.80
140	0.61	0.40	0.92
252	0.82	0.47	1.42

Table 3. Dose-response relationship between artificially sweetened beverage consumption and incident diabetic retinopathy

Dose	Exp(xb)	L95%CI	U95%CI
0	1.00	1.00	1.00
591.7999	1.28	0.95	1.73
1420.2	1.47	0.98	2.21

Table 4. Dose-response relationship between artificially sweetened beverage consumption and any diabetic retinopathy.

Dose	Exp(xb)	L95%CI	U95%CI
0	1.00	1.00	1.00
591.79999	1.28	0.77	2.13
1420.2	1.44	0.71	2.91

Table 5. Dose-response relationship between artificially sweetened beverage consumption and mild non-proliferative diabetic retinopathy.

Dose	Exp(xb)	L95%CI	U95%CI
0	1.00	1.00	1.00
591.79999	1.07	0.43	2.64
1420.2	0.45	0.08	2.45

Table 6. Dose-response relationship between artificially sweetened beverage consumption and moderate non-proliferative diabetic retinopathy.

Dose	Exp(xb)	L95%CI	U95%CI
0	1.00	1.00	1.00
591.79999	1.20	0.67	2.15
1420.2	1.28	0.57	2.88

Table 7. Dose-response relationship between artificially sweetened beverage consumption and severe non-proliferative diabetic retinopathy.

Dose	Exp(xb)	L95%CI	U95%CI
0	1.00	1.00	1.00
591.79999	0.67	0.21	2.14
1420.2	1.20	0.32	4.47

Table 8. Dose-response relationship between artificially sweetened beverage consumption and proliferative diabetic retinopathy.

Dose	Exp(xb)	L95%CI	U95%CI
0	1.00	1.00	1.00
591.79999	1.92	0.98	3.76
1420.2	2.62	1.14	6.04

Table 9. Dose-response relationship between sugar-sweetened beverage consumption and incident diabetic retinopathy.

Dose	Exp(xb)	L95%CI	U95%CI
0	1.00	1.00	1.00
98.599998	0.78	0.60	1.01
295.89999	0.92	0.69	1.23
473.39999	0.98	0.64	1.50

Table 10. Dose-response relationship between natural juice consumption and incident diabetic retinopathy.

Dose	Exp(xb)	L95%CI	U95%CI
0	1.00	1.00	1.00
98.599998	1.06	0.87	1.30
295.89999	1.03	0.72	1.50
473.39999	0.78	0.50	1.21

Table 11. Dose-response relationship between yogurt consumption and incident diabetic retinopathy.

Dose	Exp(xb)	L95%CI	U95%CI
0	1.00	1.00	1.00
98.599998	0.81	0.64	1.02
295.89999	1.11	0.87	1.42
473.39999	0.99	0.59	1.67

Table 12. Dose-response relationship between coffee consumption and incident diabetic retinopathy.

Dose	Exp(xb)	L95%CI	U95%CI
0	1.00	1.00	1.00
840	0.86	0.63	1.18
1680	0.62	0.46	0.83
2016	0.55	0.40	0.76
4032	0.42	0.26	0.68

Table 13. Dose-response relationship between coffee consumption and any diabetic retinopathy.

Dose	Exp(xb)	L95%CI	U95%CI
0	1.00	1.00	1.00
840	0.95	0.54	1.68
1680	0.67	0.36	1.24
4032	0.53	0.28	1.00

Table 14. Dose-response relationship between coffee consumption and vision-threatening diabetic retinopathy (VTDR).

Dose	Exp(xb)	L95%CI	U95%CI
0	1.00	1.00	1.00
840	1.44	0.60	3.44
1680	0.57	0.21	1.54
4032	0.30	0.10	0.90

Table 15. Dose-response relationship between coffee consumption and proliferative diabetic retinopathy.

Dose	Exp(xb)	L95%CI	U95%CI
0	1.00	1.00	1.00
840	0.73	0.20	2.63
1680	0.41	0.10	1.68
4032	0.28	0.06	1.36

Table 16. Dose-response relationship between black coffee consumption and incident diabetic retinopathy.

Dose	Exp(xb)	L95%CI	U95%CI
0	1.00	1.00	1.00
840	0.90	0.45	1.79
2016	0.57	0.34	0.95

Table 17. Dose-response relationship between coffee with sugar or cream consumption and incident diabetic retinopathy.

Dose	Exp(xb)	L95%CI	U95%CI
0	1.00	1.00	1.00
840	0.63	0.35	1.13
2016	0.55	0.29	1.05

Table 18. Dose-response relationship between tea consumption and incident diabetic retinopathy.

Dose	Exp(xb)	L95%CI	U95%CI
0	1.00	1.00	1.00
98.599998	0.81	0.62	1.06
295.89999	0.72	0.58	0.90
473.39999	0.72	0.57	0.91

Supplementary Table 5 Data used for dose-response meta-analyses

Beverage	author	year	n	cases	dose	Effect Measure	Effect estimate	95% CI lower	95% CI upper	Notes
Alcohol	C. C. Lee	2010	833	456	0	OR	1	1	1	T2DM
Alcohol	C. C. Lee	2010	316	139	266.3	OR	0.88	0.65	1.2	T2DM
Alcohol	C. C. Lee	2010	90	45	596.5	OR	1.08	0.66	1.75	T2DM
Alcohol	Preeti Gupta	2021	444	75	0	OR	1	1	1	T2DM
Alcohol	Preeti Gupta	2021	46	3	14	OR	0.17	0.04	0.69	T2DM
Alcohol	Preeti Gupta	2021	19	4	33.6	OR	1.16	0.3	4.53	T2DM
Alcohol	Bo Li	2024	1666	311	0	OR	1	1	1	T2DM
Alcohol	Bo Li	2024	1666	311	5.25	OR	0.487	0.32	0.748	T2DM
Alcohol	Bo Li	2024	158	33	25.2	OR	0.82	0.42	1.593	T2DM
Alcohol	E. Martín-Merino	2017	1051	70	4	OR	1	1	1	T2DM
Alcohol	E. Martín-Merino	2017	622	47	92	OR	1.29	0.85	1.96	T2DM
Alcohol	E. Martín-Merino	2017	103	17	211.2	OR	2.88	1.49	5.55	T2DM
Alcohol	J.W.J.Beulens•J.S	2008	254	53	0	OR	1	1	1	T1DM
Alcohol	J.W.J.Beulens•J.S	2008	345	61	2.5	OR	1.05	0.65	1.69	T1DM
Alcohol	J.W.J.Beulens•J.S	2008	390	60	17.5	OR	0.64	0.4	1.03	T1DM
Alcohol	J.W.J.Beulens•J.S	2008	332	47	50	OR	0.6	0.37	0.99	T1DM
Alcohol	J.W.J.Beulens•J.S	2008	382	55	140	OR	0.62	0.38	1.01	T1DM
Alcohol	J.W.J.Beulens•J.S	2008	154	28	252	OR	0.88	0.48	1.63	T1DM
Alcohol	Harjutsalo	2013	1690	500	42	OR	1	1	1	T1DM
Alcohol	Harjutsalo	2013	858	325	0	OR	1.42	1.11	1.82	T1DM
Alcohol	Harjutsalo	2013	141	74	0	OR	1.73	1.07	2.79	T1DM
Tea	Xiaoyu Lin	2025	1456	146	0	HR	1	1	1	/
Tea	Xiaoyu Lin	2025	1148	93	98.6	HR	0.81	0.62	1.06	/
Tea	Xiaoyu Lin	2025	2310	176	295.9	HR	0.72	0.58	0.9	/
Tea	Xiaoyu Lin	2025	1762	133	473.4	HR	0.72	0.57	0.92	/
Coffee	Hak Jun Lee	2022	231	53	0	OR	1	1	1	Any DR
Coffee	Hak Jun Lee	2022	310	61	840	OR	0.95	0.54	1.68	Any DR
Coffee	Hak Jun Lee	2022	365	72	1680	OR	0.67	0.36	1.24	Any DR
Coffee	Hak Jun Lee	2022	444	84	4032	OR	0.53	0.28	0.99	Any DR
Coffee	Hak Jun Lee	2022	231	14	0	OR	1	1	1	VTDR
Coffee	Hak Jun Lee	2022	310	18	840	OR	1.44	0.6	3.43	VTDR
Coffee	Hak Jun Lee	2022	365	23	1680	OR	0.57	0.21	1.53	VTDR
Coffee	Hak Jun Lee	2022	444	17	4032	OR	0.3	0.1	0.91	VTDR
Coffee	Hak Jun Lee	2022	231	8	0	OR	1	1	1	PDR
Coffee	Hak Jun Lee	2022	310	9	840	OR	0.73	0.20	2.60	PDR
Coffee	Hak Jun Lee	2022	365	9	1680	OR	0.41	0.1	1.67	PDR
Coffee	Hak Jun Lee	2022	444	7	4032	OR	0.28	0.06	1.42	PDR
Coffee	Hak Jun Lee	2022	933	193	0	OR	1	1	1	black coffee
Coffee	Hak Jun Lee	2022	137	27	840	OR	0.90	0.45	1.79	black coffee
Coffee	Hak Jun Lee	2022	280	50	2016	OR	0.57	0.34	0.95	black coffee
Coffee	Hak Jun Lee	2022	921	190	0	OR	1	1	1	coffee with sugar or cream intake
Coffee	Hak Jun Lee	2022	251	46	840	OR	0.63	0.35	1.13	coffee with sugar or cream intake
Coffee	Hak Jun Lee	2022	178	34	2016	OR	0.55	0.29	1.06	coffee with sugar or cream intake
ASBs	Fenwick, E. K.	2018	190	101	0	OR	1	1	1	any DR
ASBs	Fenwick, E. K.	2018	193	120	591.8	OR	1.28	0.77	2.13	any DR
ASBs	Fenwick, E. K.	2018	90	65	1420.2	OR	1.44	0.71	2.90	any DR
ASBs	Fenwick, E. K.	2018	190	29	0	OR	1	1	1	PDR
ASBs	Fenwick, E. K.	2018	193	51	591.8	OR	1.92	0.98	3.76	PDR
ASBs	Fenwick, E. K.	2018	90	35	1420.2	OR	2.62	1.14	6.06	PDR
ASBs	Fenwick, E. K.	2018	190	14	0	OR	1	1	1	Mild NPDR
ASBs	Fenwick, E. K.	2018	193	12	591.8	OR	1.07	0.43	2.62	Mild NPDR
ASBs	Fenwick, E. K.	2018	90	1	1420.2	OR	0.45	0.08	2.38	Mild NPDR
ASBs	Fenwick, E. K.	2018	190	48	0	OR	1	1	1	Moderate NPDR
ASBs	Fenwick, E. K.	2018	193	52	591.8	OR	1.2	0.67	2.15	Moderate NPDR
ASBs	Fenwick, E. K.	2018	90	23	1420.2	OR	1.28	0.57	2.88	Moderate NPDR
ASBs	Fenwick, E. K.	2018	190	10	0	OR	1	1	1	Severe NPDR
ASBs	Fenwick, E. K.	2018	193	5	591.8	OR	0.67	0.21	2.15	Severe NPDR
ASBs	Fenwick, E. K.	2018	90	6	1420.2	OR	1.2	0.32	4.44	Severe NPDR
SSB	Xiaoyu Lin	2025	4739	404	0	HR	1	1	1	/
SSB	Xiaoyu Lin	2025	1041	69	98.6	HR	0.78	0.6	1	/
SSB	Xiaoyu Lin	2025	613	51	295.9	HR	0.92	0.69	1.24	/
Natural juice	Xiaoyu Lin	2025	3864	322	0	HR	1	1	1	/
Natural juice	Xiaoyu Lin	2025	1727	144	98.6	HR	1.06	0.86	1.29	/
Natural juice	Xiaoyu Lin	2025	736	60	295.9	HR	1	0.76	1.33	/
Natural juice	Xiaoyu Lin	2025	349	22	473.4	HR	0.78	0.5	1.21	/
Yogurt	Xiaoyu Lin	2025	4119	358	0	HR	1	1	1	/
Yogurt	Xiaoyu Lin	2025	1448	96	98.6	HR	0.81	0.64	1.02	/
Yogurt	Xiaoyu Lin	2025	922	79	295.9	HR	1.11	0.87	1.42	/
Yogurt	Xiaoyu Lin	2025	187	15	473.4	HR	0.99	0.59	1.67	/

Supplementary Table 6. Subgroup analyses by specific alcoholic beverage type and diabetes type

Beverage type	Subgroup	No. of studies	OR (95% CI)	P value
Wine by type	White wine	1	0.51 (0.28–0.95)	0.035
Wine by type	Red wine	1	0.74(0.38–1.45)	0.380
Wine by type	Sherry	1	0.22 (0.05–0.95)	0.042
Wine by type	General wine	1	2.32 (1.35–3.99)	0.002
Spirits by diabetes type	Type 1 diabetes	1	2.32 (1.35–3.99)	0.002
Spirits by diabetes type	Type 2 diabetes	1	1.28(0.62–2.65)	0.505

Abbreviations: CI, confidence interval; DR, diabetic retinopathy; NS, not significant; OR, odds ratio.

Supplementary Table 7-1. Alcohol intake and risk of diabetic retinopathy occurrence and progression: GRADE evidence profile

Certainty assessment	No. of studies	Study design	Risk of bias	Inconsistency	Indirectness	Imprecision	Publication bias	Effect size (95% CI)	Overall certainty
any DR (occurrence)	21	Cohort/case-control/cross-sectional	Not serious[1]	Not serious[2]	Not serious	Not serious	Undetected[3]	OR 0.92 (0.80–1.05)	⊕⊕◯◯ Low
NPDR (occurrence)	1	Cross-sectional	Not serious	Not serious	Not serious	Serious[4] (−1)	Undetected[3]	OR 0.81 (0.69–0.95)	⊕◯◯◯ Very low
VTDR (occurrence)	2	Cohort/cross-sectional	Not serious	Not serious	Not serious	Serious[4] (−1)	Undetected[3]	OR 5.13 (1.96–13.43)	⊕⊕◯◯ Low
DR (progression)	3	Cohort	Not serious	Serious[5] (−1)	Not serious	Not serious	Undetected[3]	OR 1.27 (0.68,2.37)	⊕◯◯◯ Very low

Footnotes:

[1] The majority of included studies were rated as high quality (NOS score ≥ 7), and no serious risk of bias was identified.

[2] I^2 = 62.8% for this analysis; however, subgroup analyses stratified by region and diabetes type showed a consistent direction of association across most subgroups. Therefore, the evidence was not downgraded for inconsistency.

[3] Publication bias was assessed using funnel plots and Egger's test for analyses including ≥ 10 studies, with no obvious asymmetry detected; for analyses with fewer than 10 studies, no formal assessment for publication bias was conducted.

[4] Downgraded one level for imprecision: the analysis was based on a single study or a limited number of participants/events, with wide confidence intervals that crossed the null line and/or boundaries of minimal clinically important difference.

[5] Downgraded one level for inconsistency: I^2 = 68.2%, with substantial variation in point estimates across studies. The small number of studies precluded full exploration of heterogeneity sources through subgroup analysis or meta-regression.

Absolute effect estimates were not calculated owing to substantial variation in baseline DR risk across the included populations and the diversity of study designs.

Supplementary Table 7-2. Beverage intake and dose-response relationships with diabetic retinopathy: GRADE evidence profile

Certainty assessment	No. of studies	Study design	Risk of bias	Inconsistency	Indirectness	Imprecision	Publication bias	Dose-response evidence	Overall certainty
Tea → DR	2	Case-control/cohort	Not serious	Not serious	Serious[1] (−1)	Not serious	Undetected[2]	Non-linear; increasing intake associated with lower risk (+1)	⊕⊕⊕◯ Moderate
Coffee → DR	1	Cross-sectional	Not serious	Not serious	Not serious	Serious[3] (−1)	Undetected[2]	Non-linear; more pronounced inverse association at highest intake levels (+1)	⊕⊕◯◯ Low
Coffee → VTDR	1	Cross-sectional	Not serious	Not serious	Not serious	Serious[3] (−1)	Undetected[2]	Non-linear; high intake significantly associated with lower risk (+1)	⊕⊕◯◯ Low
ASBs → PDR	1	Cross-sectional	Not serious	Not serious	Not serious	Serious[3] (−1)	Undetected[2]	Non-linear; increasing intake associated with higher risk (+1)	⊕⊕◯◯ Low
Black coffee → DR	1	Cross-sectional	Not serious	Not serious	Not serious	Serious[3] (−1)	Undetected[2]	Non-linear; moderate to high intake associated with lower risk (+1)	⊕⊕◯◯ Low
SSBs → DR	1	Cohort	Not serious	Not serious	Not serious	Serious[3] (−1)	Undetected[2]	None identified	⊕◯◯◯ Very low

Certainty assessment	No. of studies	Study design	Risk of bias	Inconsistency	Indirectness	Imprecision	Publication bias	Dose-response evidence	Overall certainty
Natural fruit juice → DR	1	Cohort	Not serious	Not serious	Not serious	Serious[3] (−1)	Undetected[2]	None identified	⊕◯◯◯ Very low
Yogurt → DR	1	Cohort	Not serious	Not serious	Not serious	Serious[3] (−1)	Undetected[2]	None identified	⊕◯◯◯ Very low

Footnotes:

[1] Downgraded one level for indirectness: the current evidence is predominantly derived from Asian populations, limiting direct generalizability to other ethnic and geographic groups.

[2] All analyses included fewer than 10 studies; therefore, no formal assessment for publication bias was conducted.

[3] Downgraded one level for imprecision: the analysis was based on a single study or a limited number of participants/events, with wide confidence intervals that crossed the null line and/or boundaries of minimal clinically important difference.

Absolute effect estimates were not calculated owing to substantial variation in baseline DR risk across the included populations and the diversity of study designs.

Supplementary Table 7-3. Specific alcoholic beverage types and risk of diabetic retinopathy: GRADE evidence profile

Certainty assessment	No. of studies	Study design	Risk of bias	Inconsistency	Indirectness	Imprecision	Publication bias	Effect size (95% CI)	Overall certainty
Wine → DR	2	Cross-sectional	Not serious	Serious[1] (−1)	Not serious	Not serious	Undetected[2]	OR 1.08 (0.58–2.03)	⊕◯◯◯ Very low
White wine → DR	1	Cross-sectional	Not serious	Not serious	Not serious	Serious[3] (−1)	Undetected[2]	OR 0.51 (0.28–0.95)	⊕◯◯◯ Very low
Sherry → DR	1	Cross-sectional	Not serious	Not serious	Not serious	Serious[3] (−1)	Undetected[2]	OR 0.22 (0.05–0.95)	⊕◯◯◯ Very low
General wine → DR	1	Cross-sectional	Not serious	Not serious	Not serious	Serious[3] (−1)	Undetected[2]	OR 2.32 (1.35–3.99)	⊕◯◯◯ Very low
Spirits → DR	2	Cross-sectional	Not serious	Not serious	Not serious	Not serious	Undetected[2]	OR 1.87 (1.21–2.90)	⊕ ⊕◯◯ Low

Footnotes:

[1] Downgraded one level for inconsistency: I^2 = 75.9%, with two studies showing opposite directions of effect (one inverse, one positive), and clear differences in the definition and classification of wine across studies.

[2] All analyses included fewer than 10 studies; therefore, no formal assessment for publication bias was conducted.

[3] Downgraded one level for imprecision: the analysis was based on a single study, with wide confidence intervals that crossed the null line and/or boundaries of minimal clinically important difference.

Absolute effect estimates were not calculated owing to substantial variation in baseline DR risk across the included populations and the diversity of study designs.

Supplementary Figure 1. Analysis of the association between alcohol consumption and any diabetic retinopathy. (A) Funnel plot for publication bias assessment. (B) Sensitivity analysis (leave-one-out method). (C) Subgroup analysis stratified by region. (D) Subgroup analysis stratified by study design. (E) Subgroup analysis stratified by sample size. Pooled estimates were obtained using random-effects models. Effect estimates are expressed as odds ratios with 95% confidence intervals. CI, confidence interval; DR, diabetic retinopathy; OR, odds ratio.

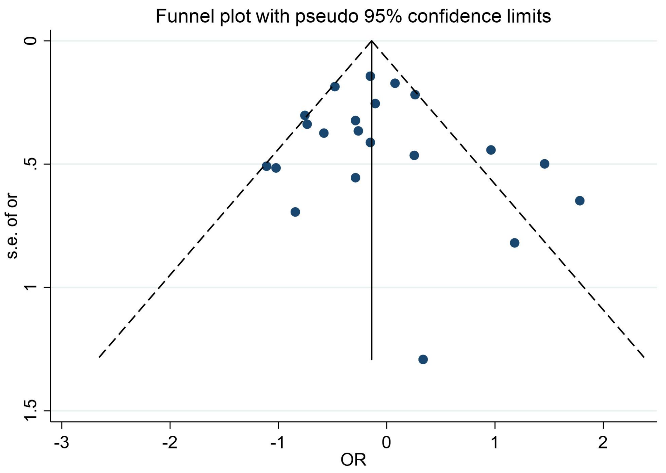

（A）

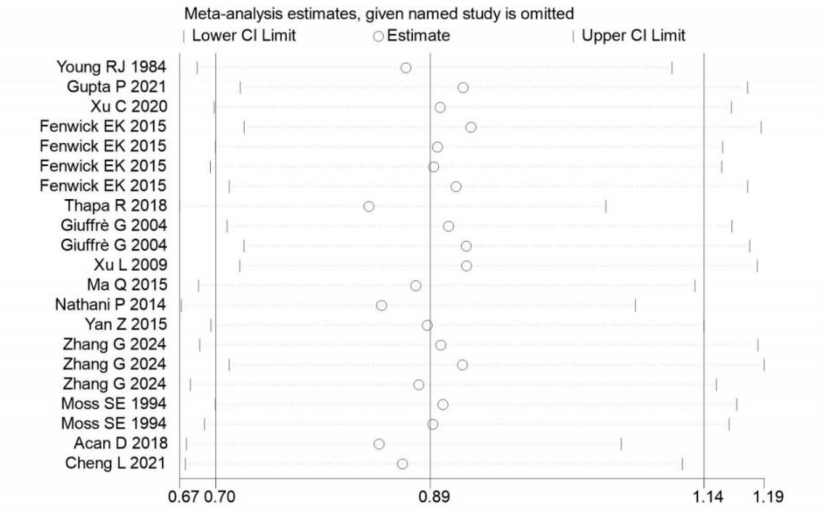

（B）

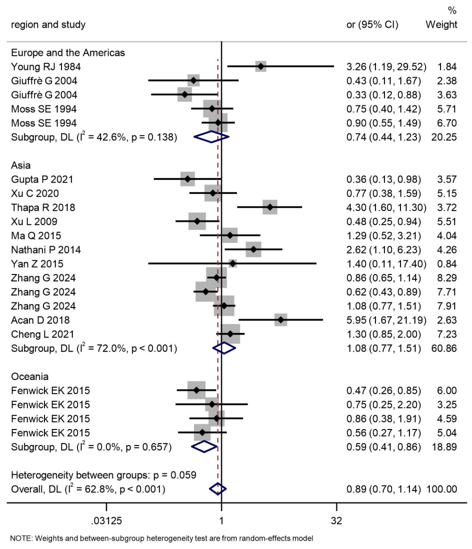

（C）

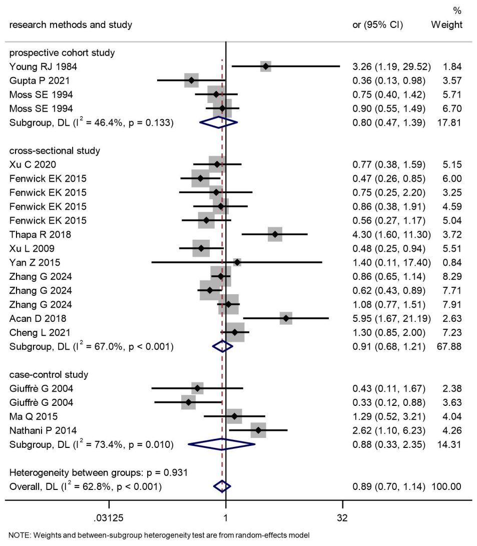

（D）

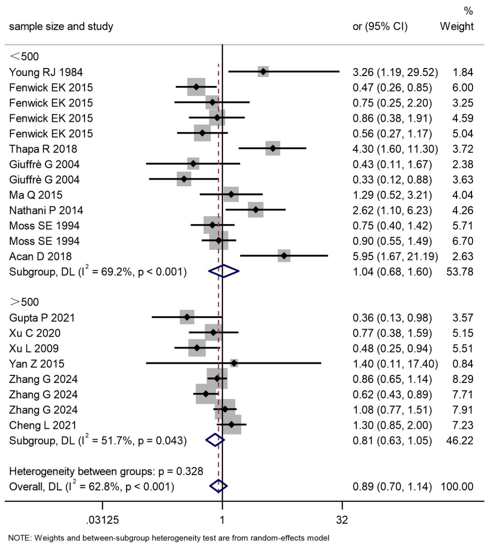

（E）

Supplementary Figure 2. Analysis of the association between alcohol consumption and non-proliferative diabetic retinopathy. (A) Sensitivity analysis (leave-one-out method). (B) Subgroup analysis stratified by diabetes type. Pooled estimates were obtained using a fixed-effect model. Effect estimates are expressed as odds ratios with 95% confidence intervals. Publication bias was not assessed due to the limited number of included studies (<10). CI, confidence interval; DR, diabetic retinopathy; NPDR, non-proliferative diabetic retinopathy; OR, odds ratio; T1DM, type 1 diabetes mellitus; T2DM, type 2 diabetes mellitus.

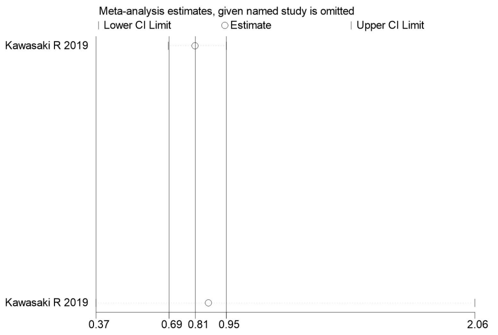

（A）

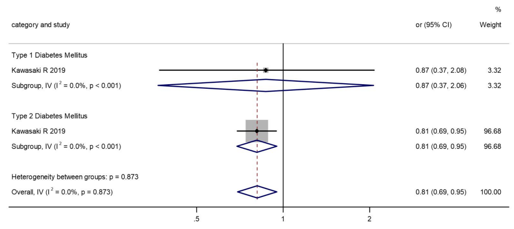

（B）

Supplementary Figure 3. Analysis of the association between alcohol consumption and vision-threatening diabetic retinopathy. (A) Sensitivity analysis (leave-one-out method). (B) Subgroup analysis stratified by study design. Pooled estimates were obtained using a fixed-effect model. Effect estimates are expressed as odds ratios with 95% confidence intervals. Publication bias was not assessed due to the limited number of included studies (<10). CI, confidence interval; DR, diabetic retinopathy; OR, odds ratio; VTDR, vision-threatening diabetic retinopathy.

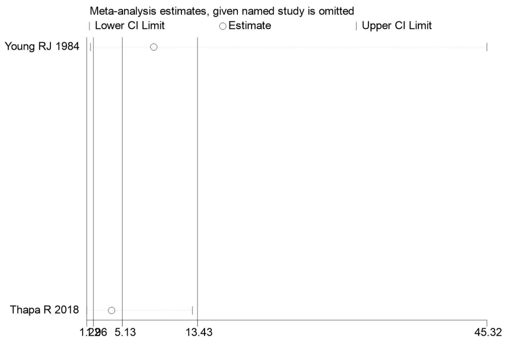

（A）

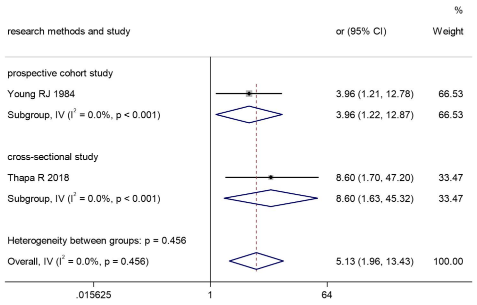

（B）

Supplementary Figure 4. Analysis of the association between alcohol consumption and progression of any diabetic retinopathy. (A) Sensitivity analysis (leave-one-out method). (B) Subgroup analysis stratified by sample size. Pooled estimates were obtained using random-effects models. Effect estimates are expressed as odds ratios with 95% confidence intervals. Publication bias was not assessed due to the limited number of included studies (<10). CI, confidence interval; DR, diabetic retinopathy; OR, odds ratio.

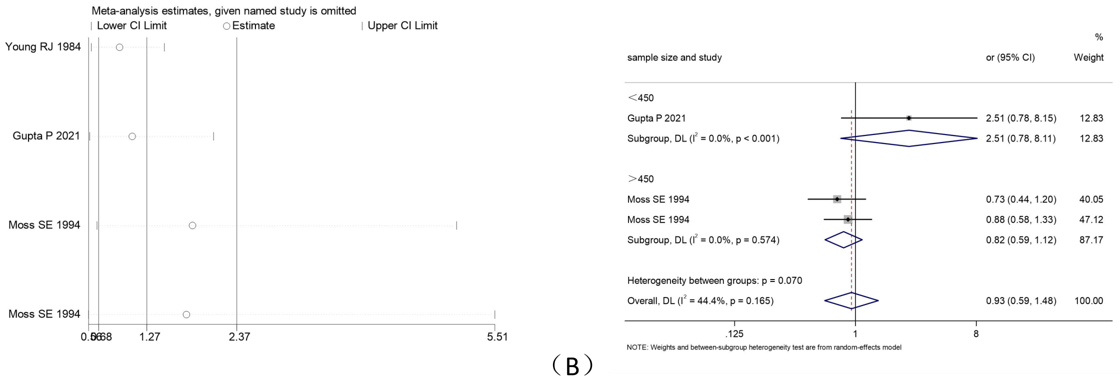

（A） （B）

Supplementary Figure 5. Analysis of the association between wine consumption and diabetic retinopathy. (A) Sensitivity analysis (leave-one-out method). (B) Subgroup analysis stratified by diabetes type. Pooled estimates were obtained using random-effects models. Effect estimates are expressed as odds ratios with 95% confidence intervals. Publication bias was not assessed due to the limited number of included studies (<10). CI, confidence interval; DR, diabetic retinopathy; OR, odds ratio.

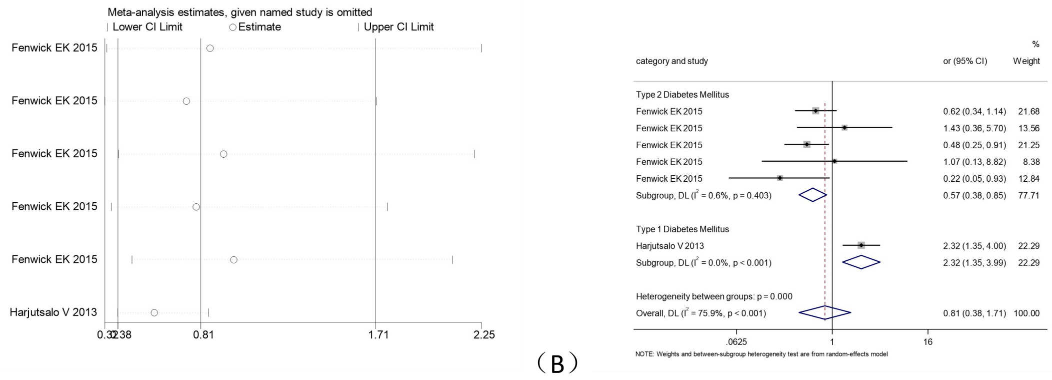

（A） （B）

Supplementary Figure 6. Analysis of the association between spirits consumption and diabetic retinopathy. (A) Sensitivity analysis (leave-one-out method). (B) Subgroup analysis stratified by diabetes type. Pooled estimates were obtained using fixed-effect models. Effect estimates are expressed as odds ratios with 95% confidence intervals. Publication bias was not assessed due to the limited number of included studies (<10). CI, confidence interval; DR, diabetic retinopathy; OR, odds ratio.

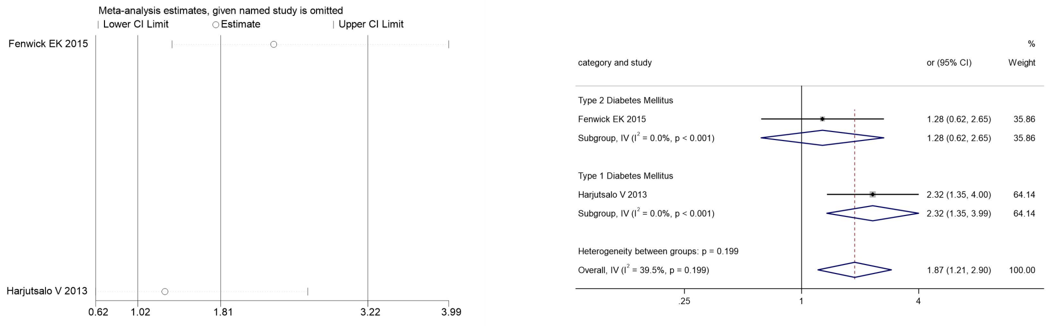

（A） （B）

Supplementary Figure 7. Analysis of the association between tea consumption and diabetic retinopathy. (A) Sensitivity analysis (leave-one-out method). (B) Subgroup analysis stratified by timing of consumption (current vs. ever). Pooled estimates were obtained using a fixed-effect model. Effect estimates are expressed as odds ratios with 95% confidence intervals. Publication bias was not assessed due to the limited number of included studies (<10). CI, confidence interval; DR, diabetic retinopathy; OR, odds ratio.

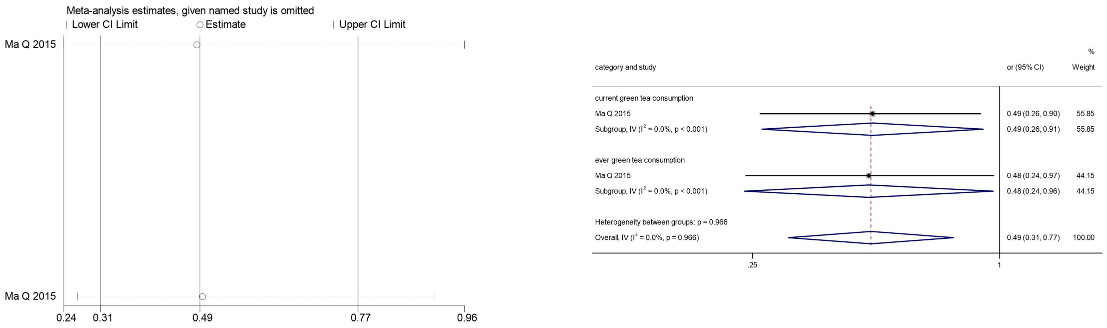

（A） （B）
